# Supplementary material for: Bioengineered niches that recreate physiological extracellular matrix organisation to support long-term haematopoietic stem cells
Source: Nat Commun. 2024 Jul 10;15:5791. doi: 10.1038/s41467-024-50054-0 (PMC11237034; doi:10.1038/s41467-024-50054-0)
Supplement: Supplementary file 1 — Supplementary Information [file 41467_2024_50054_MOESM1_ESM.pdf]

Supplementary data for:

Bioengineered niches that recreate physiological extracellular matrix organisation to support long-term haematopoietic stem cells

Hannah Donnelly[1], Ewan Ross[1], Yinbo Xiao[1], Rio Hermantara[2], Aqeel Taqi[2], W. Sebastian Doherty-Boyd[1], Jennifer Cassels[3], Penelope. M. Tsimbouri[1], Karen M Dunn[3], Jodie Hay[3], Annie Cheng[4], R.M. Dominic Meek[5], Nikhil Jain[6], Christopher West[7], Helen Wheadon[3], Alison M Michie[3], Bruno Peault[7], Adam G. West[2], Manuel Salmeron-Sanchez[4*], Matthew J. Dalby[1*].

[1]Centre for the Cellular Microenvironment, School of Molecular Biosciences, The Advanced Research Centre, 11 Chapel Lane, University of Glasgow, Glasgow, G11 6EW, United Kingdom.

[2]School of Cancer Sciences, Wolfson Wohl Cancer Research Centre, University of Glasgow, Glasgow, G61 1QH, United Kingdom.

[3]School of Cancer Sciences, Paul O'Gorman Leukaemia Research Centre, Gartnavel General Hospital, University of Glasgow, Glasgow G12 0YN, United Kingdom.

[4]Centre for the Cellular Microenvironment, Division of Biomedical Engineering, James Watt School of Engineering, The Advanced Research Centre, 11 Chapel Lane, University of Glasgow, Glasgow, G11 6EW, United Kingdom.

[5]Department of Trauma and Orthopaedics, Queen Elizabeth University Hospital, Glasgow, G51 4TF, United Kingdom.

[6]Institute of Inflammation and Ageing, University of Birmingham, Queen Elizabeth Hospital, Birmingham, B15 2WB, United Kingdom.

[7]MRC Centre for Regenerative Medicine, The University of Edinburgh, Edinburgh, EH16 4UU, United Kingdom.

* Corresponding authors:

matthew.dalby@glasgow.ac.uk

manuel.salmeron-sanchez@glasgow.ac.uk

Supplementary Figures

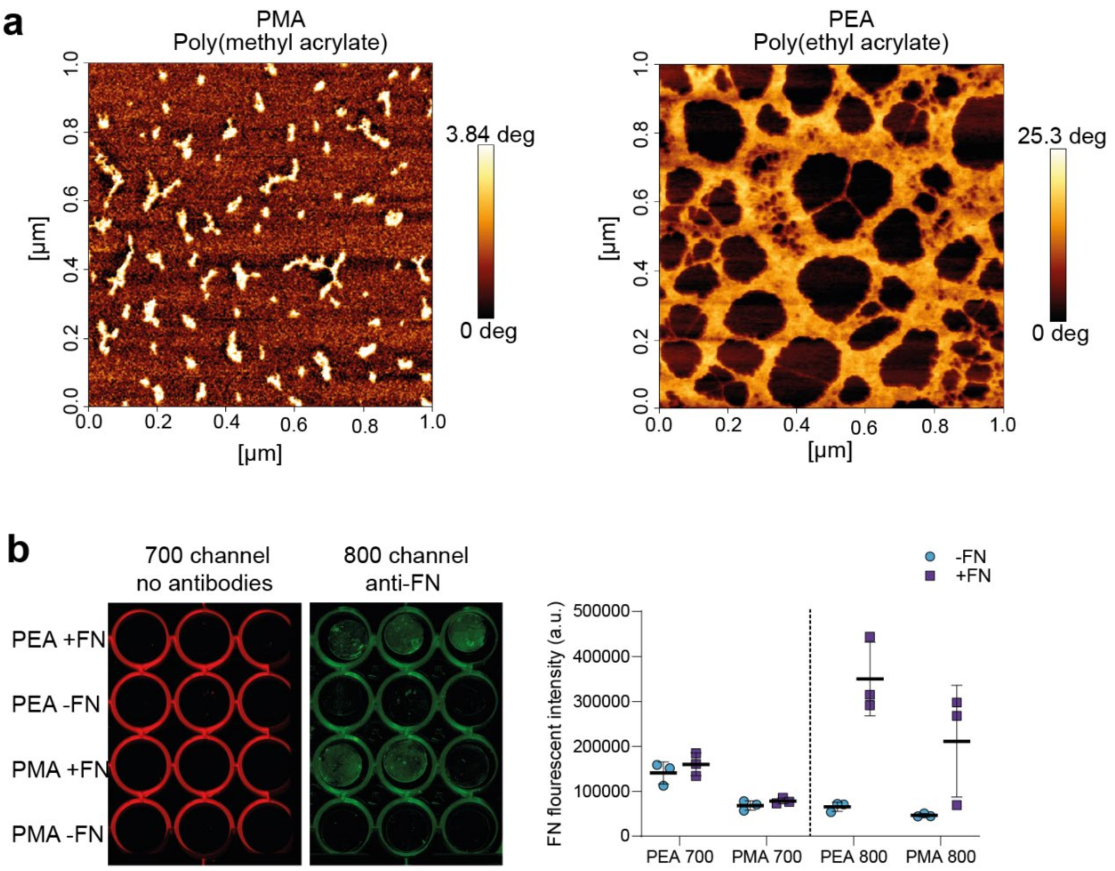

Supplementary Figure 1 | Fibronectin (FN) quantification. **a**, Representative phase images for AFM corresponding to Figure 2b. **b**, immunofluorescence analysis of PEA/PMA background fluorescence and FN quantification using in-cell western, demonstrates minimal background fluorescence observed in 700 channel containing no secondary antibodies, and 800 channel where primary anti-FN antibodies demonstrate minimal background fluorescence when there is no FN (-FN) coating on materials. Graph shows mean ±SD, blue circles = -FN, purple squares = +FN, n=3 material replicates.

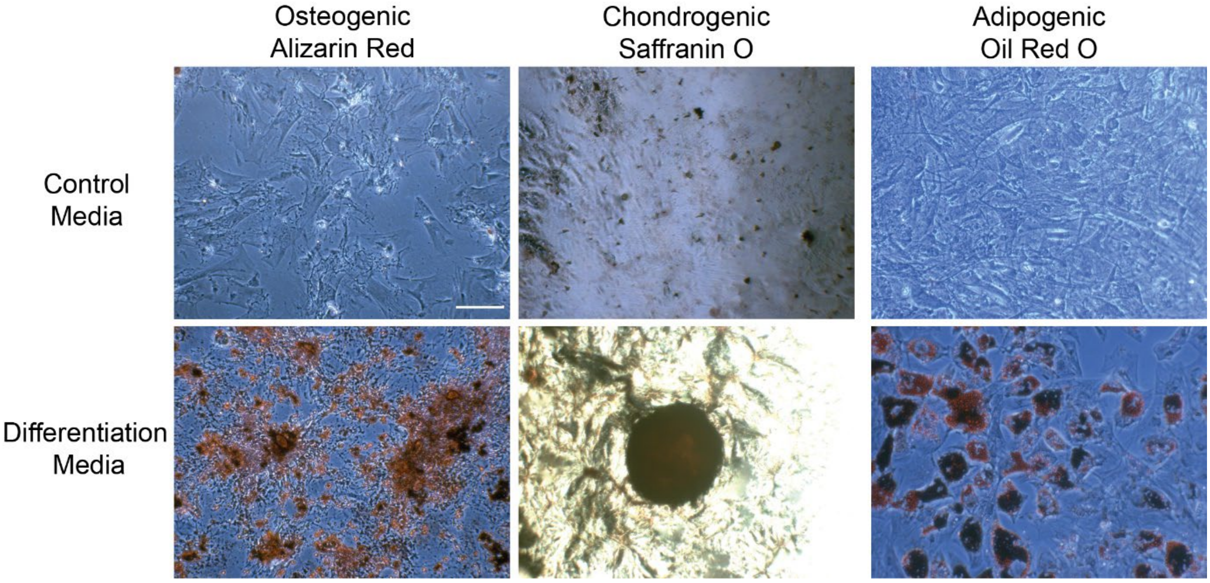

Supplementary Figure 2 | Trilineage differentiation potential of PerSCs. PerSCs were isolated and cultured ≤passage 5, then cultured in either control media or medias for osteogenic, chondrogenic and adipogenic differentiation of stromal stem cells. Histological stains were then used to assess differentiation after 4 wk culture. Alazarin red stains calcium deposits in osteogenic cultures. Safranin O is used to identify chondrocytes. Oil red O stains lipids in adipogenic cultures. Positive detection of all stains in differentiation medias demonstrates trilineage potential of PerSCs. Scale bar is 100 µm.

a

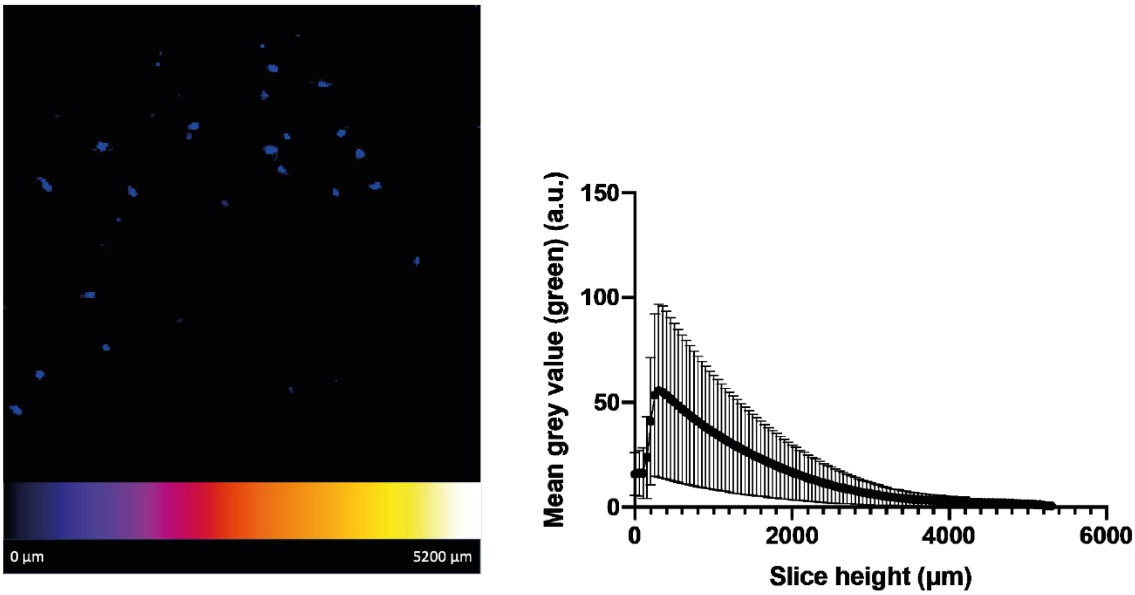

b

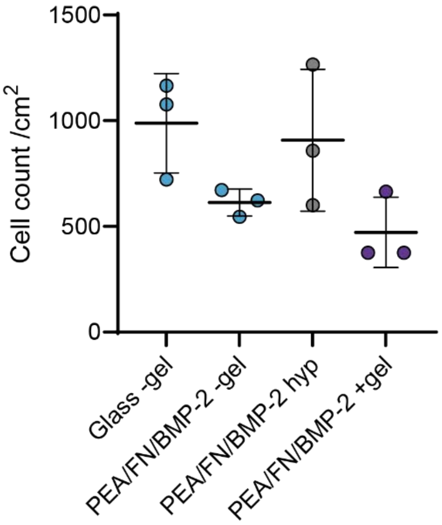

Supplementary Figure 3 | PerSC quantification. a, PerSC migration into collagen gels was assessed via Hoescht staining of PerSCs in the PEA +gel niche model. Z-projection shows minimal migration into the gel, with most cells remaining in the bottom 2000 µm of the well**. b,** Cell number quantification, via Hoescht staining. Statistics by one-way ANOVA followed by Bonferroni multiple comparison test show no significant differences. Graph shows mean ± SD, n= 3 material replicates.

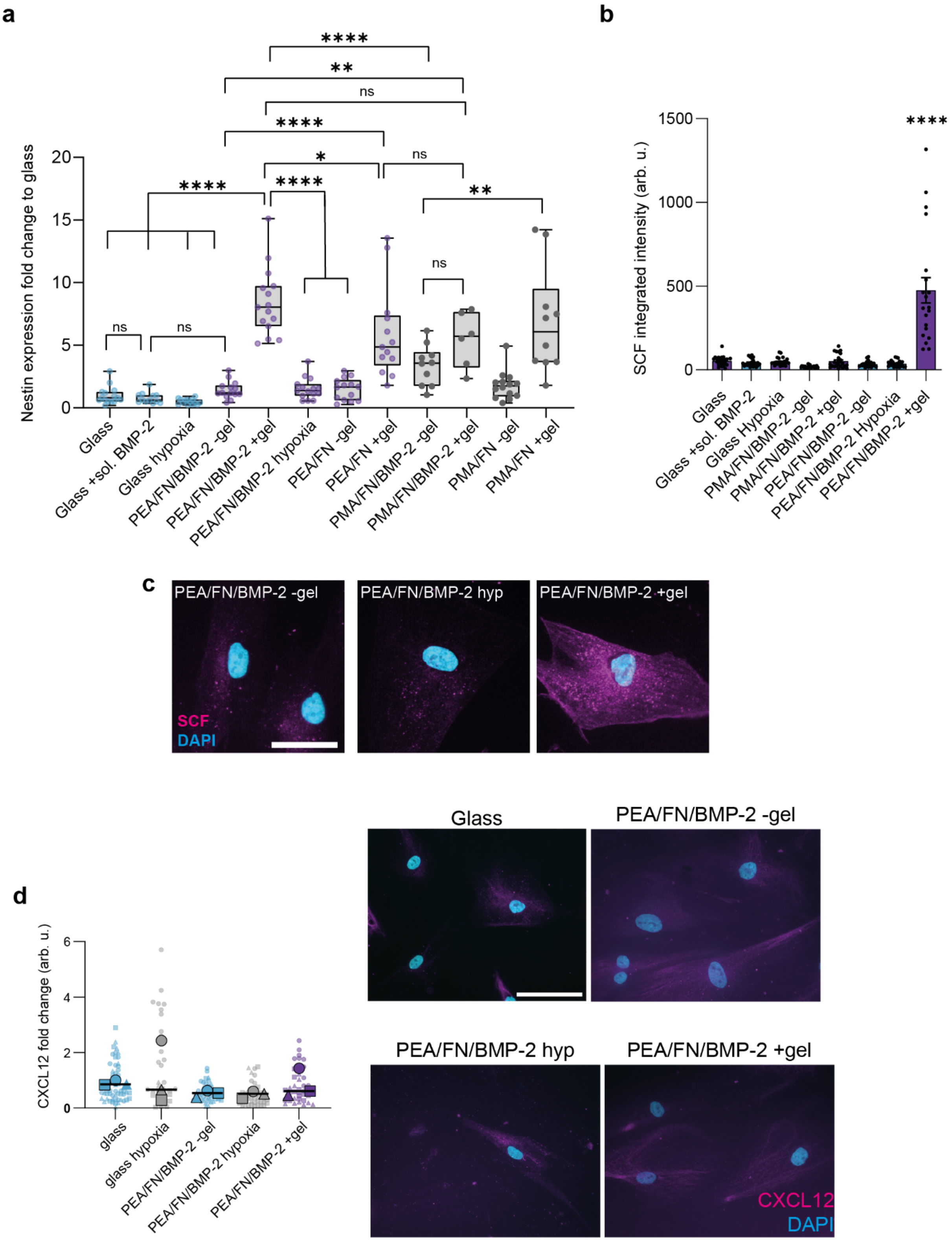

Supplementary Figure 4 | Perivascular stromal cell niche phenotype. a, Nestin expression measured by immunofluorescence at day 14 of culture. Nestin expression significantly increased in perivascular stromal cells on the PEA/FN/BMP-2 system only in the presence of low stiffness gels (+gel niche). PMA is used as a control polymer and shows increase with gel only by day 14. Glass with soluble BMP-2 (50 ng/ml) added to the media is used as a further control and shows the addition of soluble, not matrix bound, BMP-2 does not induce nestin expression. Graph shows mean integrated intensity as fold change to glass control ± SD, ****= p<0.001, **= p<0.005, determined by one-way ANOVA followed by Bonferroni multiple comparison test. Each point

represents one field of view normalised to cell number, over n= 4 materials replicates. We note a small trend of increase as we switch BMP-2 from soluble (glass +sol. BMP-2) to solid-phase (PEA-FN-BMP-2 -gel). **b, c,** SCF and **d**, CXCL12 production in niche models. Cells were cultured in niche systems for 14 days with brefeldin A treatment (5 μg/ml in culture media) added for the final 24 h to inhibit intracellular protein transport. Quantification of immunofluorescence staining with b, anti-SCF shows significant increase only in the PEA/FN/BMP-2 +gel condition. Graph shows mean ± SEM, ****= $p<0.001$, determined by one-way ANOVA followed by Bonferroni's multiple comparison test. Each point represents integrated SCF intensity of 1ximage field normalised to cell number, over n= 4 material replicates. **c**, shows representative immunofluorescence images for SCF, corresponding the B and Figure 3e. For **d**, all conditions expressed detectable CXCL12, but with no significant differences. Graph shows mean integrated intensity CXCL12 as fold change to glass control (n=3 material replicates, for 3 independent experiments with different donor cells), each point represents 1x image field normalised to cell number with background correction. Representative images from 1 donor shown, scale bar is 100 µm, magenta = CXCL12, cyan = DAPI. Non-significant, determined by one-way ANOVA followed by Bonferroni's multiple comparison test.

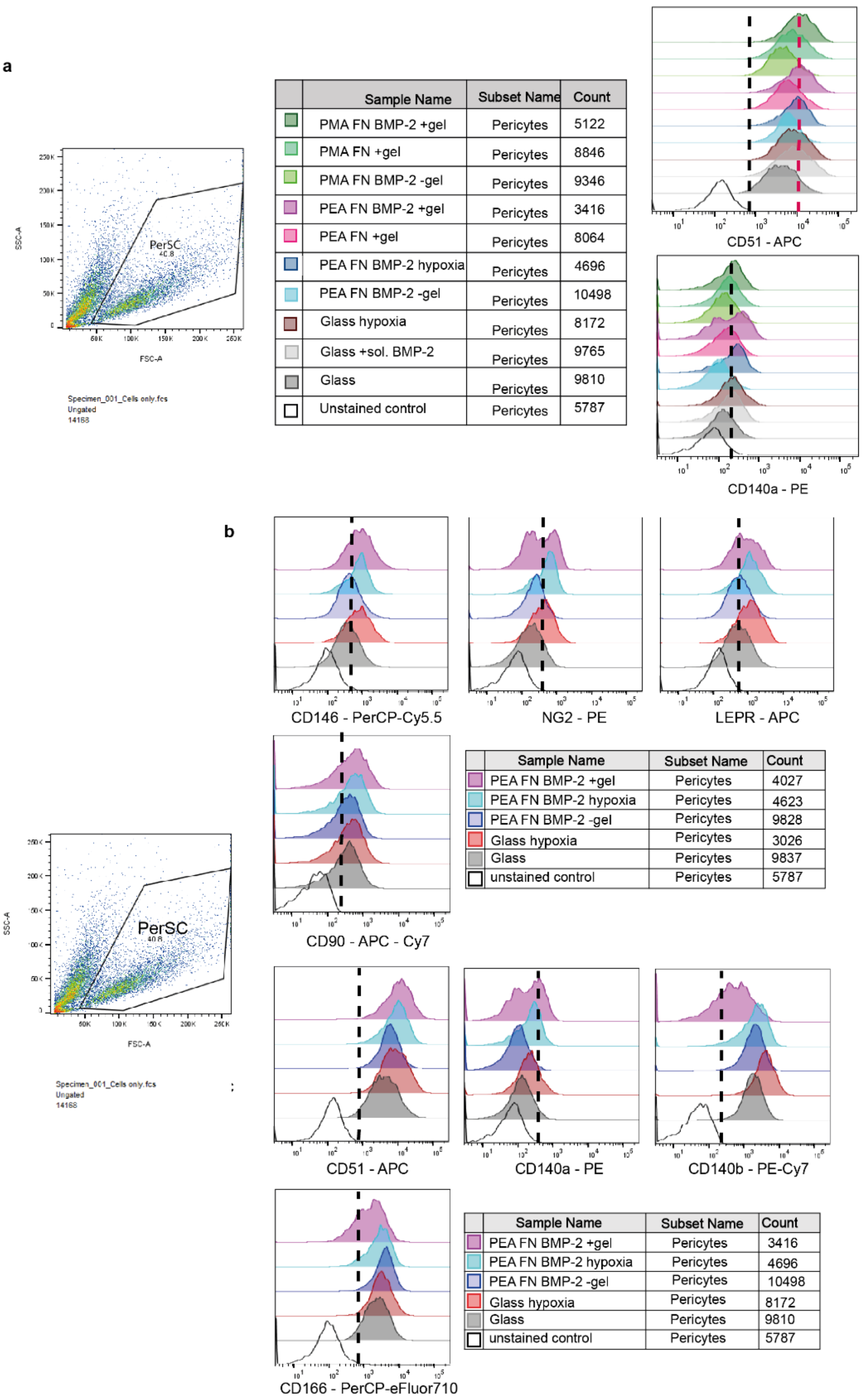

Supplementary Figure 5 | Histograms of cell surface marker phenotyping. Corresponding to Figure 3f. **a**, CD51 and CD140a – pseudo-markers for nestin expression[3] – are increased and show a double population (respectively) in nestin^{high} PerSCs in PEA/FN/BMP-2 +gel niches . **b**, shows representative histograms from one representative biological replicate for the heatmap in Figure 3f. n= 6 pooled material replicates with 1 donor represented in the histograms.

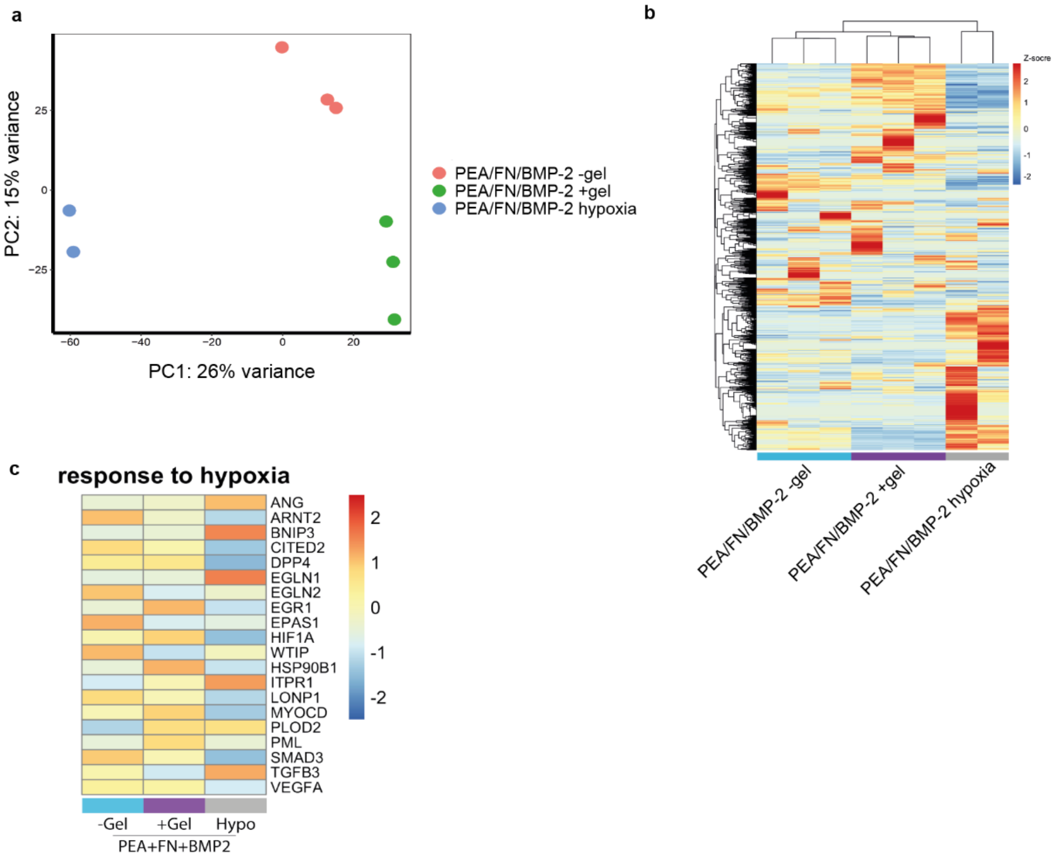

Supplementary Figure 6 | RNA-seq analysis of PerSC niche phenotype at day 7. a, Principal component analysis (PCA) shows transcripts from conditions PEA/FN/BMP-2 -gel/+gel/hypoxia samples cluster independently. **b**, Heatmap of z-scored changes in transcript levels. **c**, z-scored changes in transcript levels of genes involved in GO enrichment pathway 'response to hypoxia'. Genes with FDR < = 0.05 were considered as differentially enriched. N = 3 material replicates, for hypoxia n=2 material replicates as one replicate failed RNA quality control, from one biological donor.

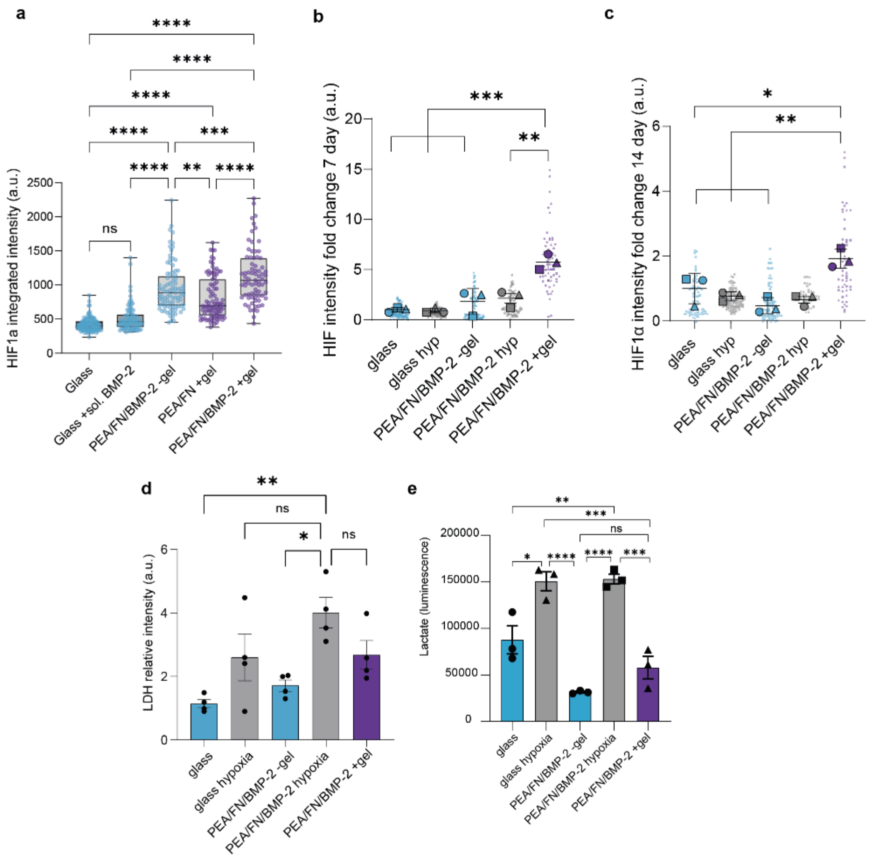

Supplementary Figure 7| HIF1a co-localisation and downstream lactate regulation. a, HIF1a co-localisation to nucleus, comparison to additional controls PEA/FN +gel and glass +soluble BMP-2, shows PEA/FN/BMP-2 +gel leads to significantly higher levels of nuclear HIF1a at 3 days. Data from one experimental repeat that is included in Figure 5a, each point represents 1x field of view normalised to cell number from n = 3 material replicates. We note a significant increase as we switch BMP-2 from soluble (glass +sol. BMP-2) to solid-phase (PEA-FN-BMP-2 -gel). **b** and **c** Nuclear HIF1α levels were compared at day 7 and 14 by immunofluorescence microscopy. HIF1α levels increased in cells in all models, and significantly increased in +gel niches. Each point represents 1 nuclei measurement with shape corresponding to mean for n = 3 material replicates from one biological donor. Graph shows means ± SEM. **d** and **e** Lactate dehydrogenase (LDH) and lactate levels are not raised with PEA/FN/BMP-2 +gel. **d**, in-cell western analysis of LDH levels in cells detected by immunofluorescence at day 7. PerSCs in PEA/FN/BMP-2 with hypoxia had significantly increased levels of the enzyme. Graph shows fluorescent intensity of LDH normalised to cell number (CellTag800) ± SEM, n = 4 material replicates. **d**, Lactate levels in cell supernatant at day 14 measured by luminescence. Glass and PEA/FN/BMP-2 hypoxia samples had significantly raised levels. Graph shows n= 3 material replicates, ± SEM. All statistics: *= p<0.05, **= p<0.005, ***= p<0.001, ****= p<0.001 determined by one-way ANOVA followed by Bonferroni's multiple comparison test.

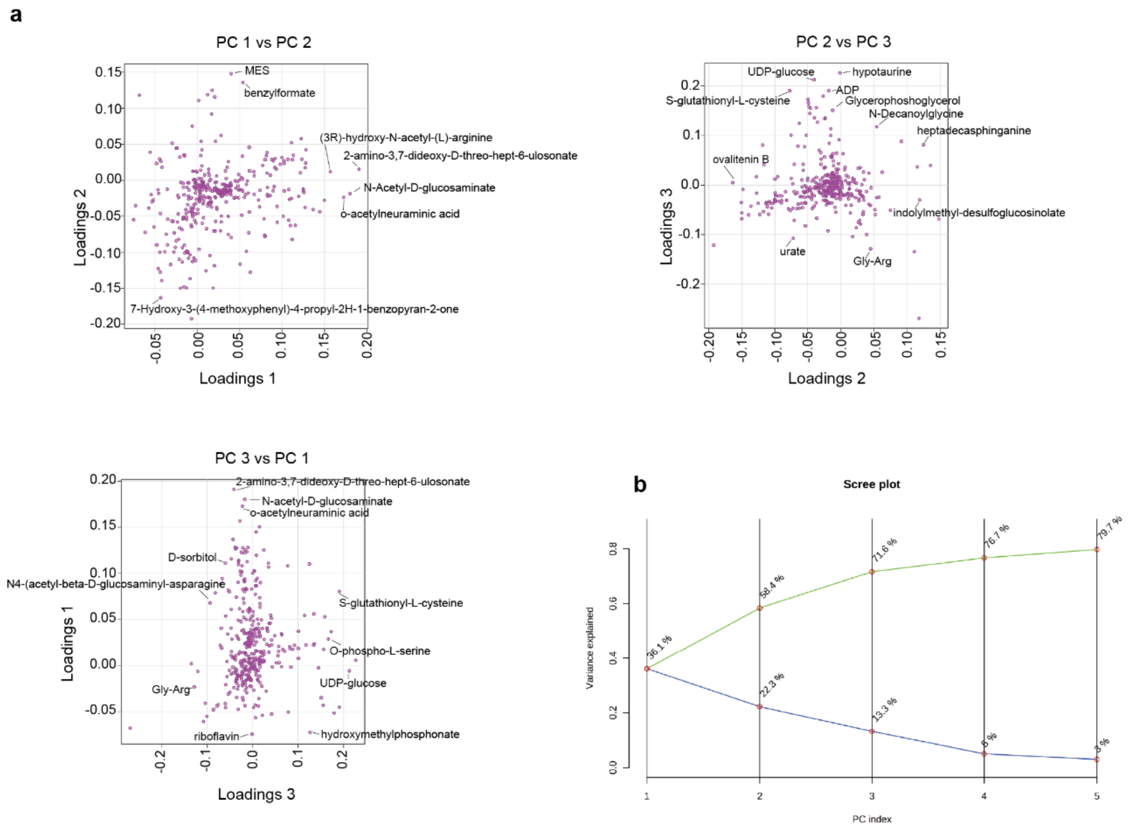

Supplementary Figure 8 | Metabolic variables in principle components. **a**, Loading plots for PCA of all metabolites with most variable metabolites labelled. **b**, Scree plot shows variance for each principal component.

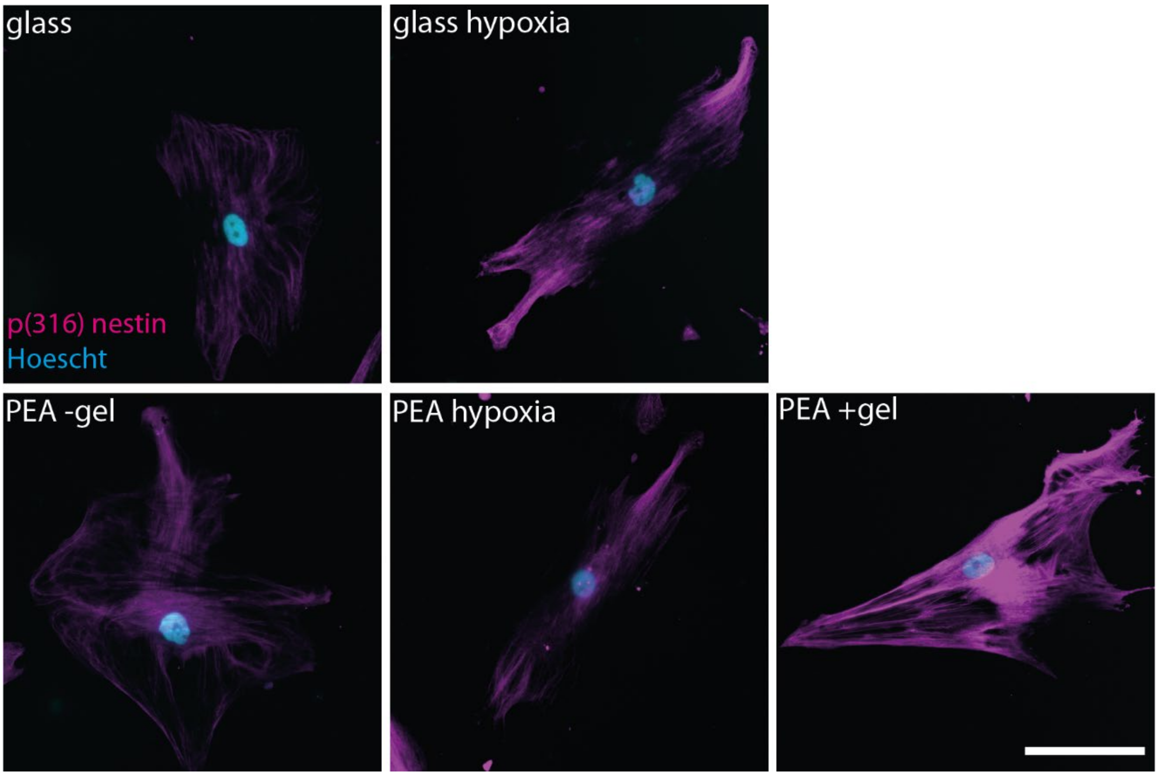

Supplementary Figure 9 | Immunofluorescent detection of nestin phosphorylation at Th(316). Representative images for Figure 6a. Suggests some nestin is phosphorylated and therefore soluble. Scale bar = 100 µm, magenta = p(316) nestin, cyan = Hoescht, n= 4 material replicates for one biological donor.

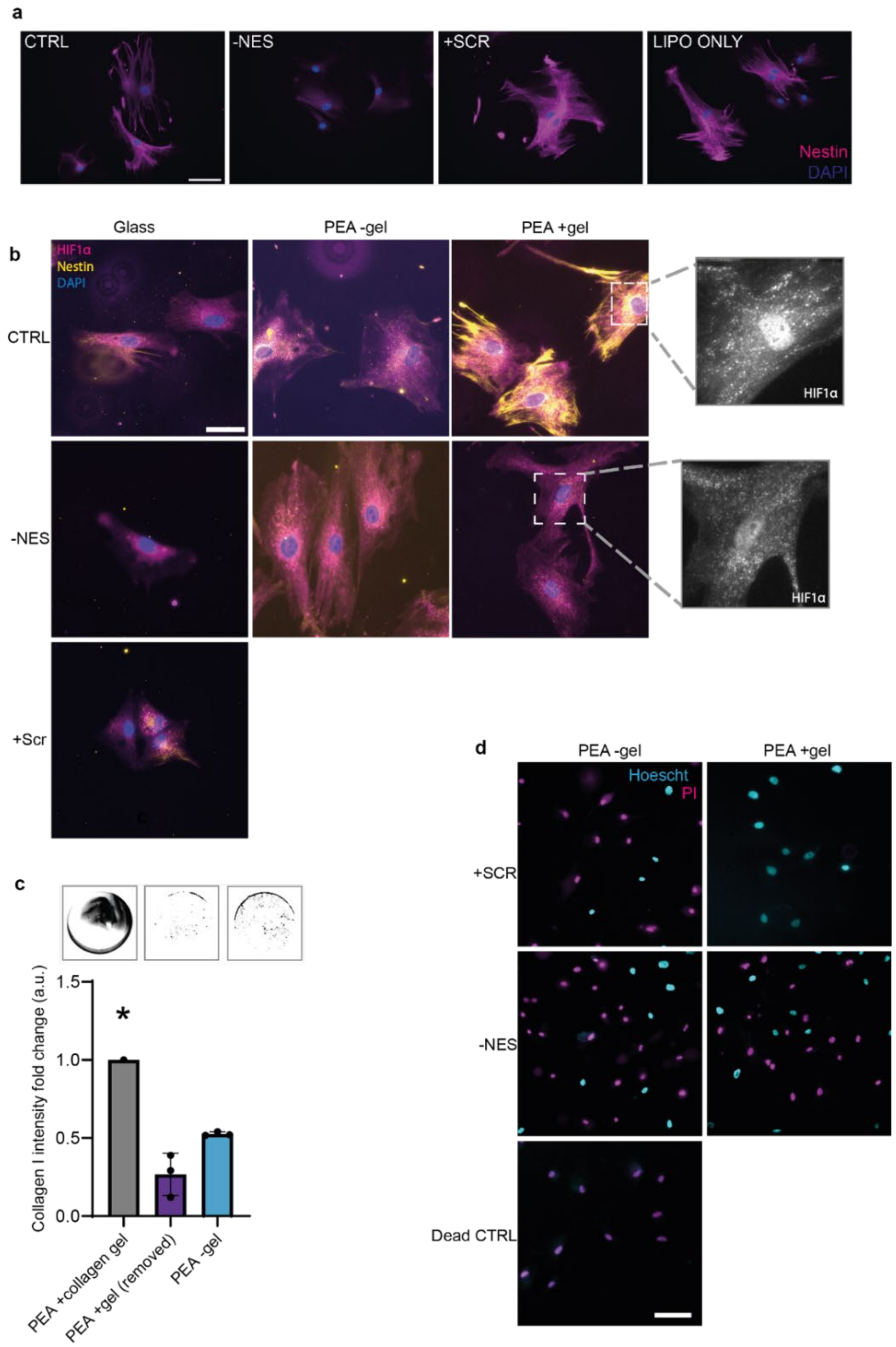

Supplementary Figure 10 | Nestin knock-down by siRNA. **a**, representative images for Figure 6b, demonstrating siRNA knock-down of nestin in PerSCs. Scale bar = 100 µm, magenta = nestin, blue = DAPI. Scr = scrambled control; lipo only = lipofectamine only control, n=3 material replicates from one biological donor**. b**, Nestin silencing leads to loss of HIF1α nuclear co-localisation in PerSCs cultured in PEA/FN/BMP-2 +gel niches. Representative images for Figure 6c. Scale bar = 100 µm, magenta = HIF1α, yellow = nestin, blue = DAPI. Representative images from one biological donor, n = 4 material replicates. **c**, Collagen I staining on fixed (but not permeabilised) PEA +PerSC coverslips after 7 days culture. Fluorescence intensity demonstrates mechanical gel removal for cytotoxicity experiments is complete and does not leave residual collagen. PEA +collagen gel (without removal) was used as a positive control. Graph shows intensity fold change to PEA -gel, mean ±SD, statistics by two-tailed students t tests (Mann Whitney), * = *p*<0.05 **d**, siRNA knock-down of nestin in PerSCs and susceptibility to oxidative stress was assessed. Representative images of 6d. All conditions were +H_2O_2, dead CTRL +10 mM H_2O_2. N= 4 material replicates, scale bar = 100 µm. Cyan = Hoescht (live and dead nuclei stain), magenta = PI (dead nuclei stain).

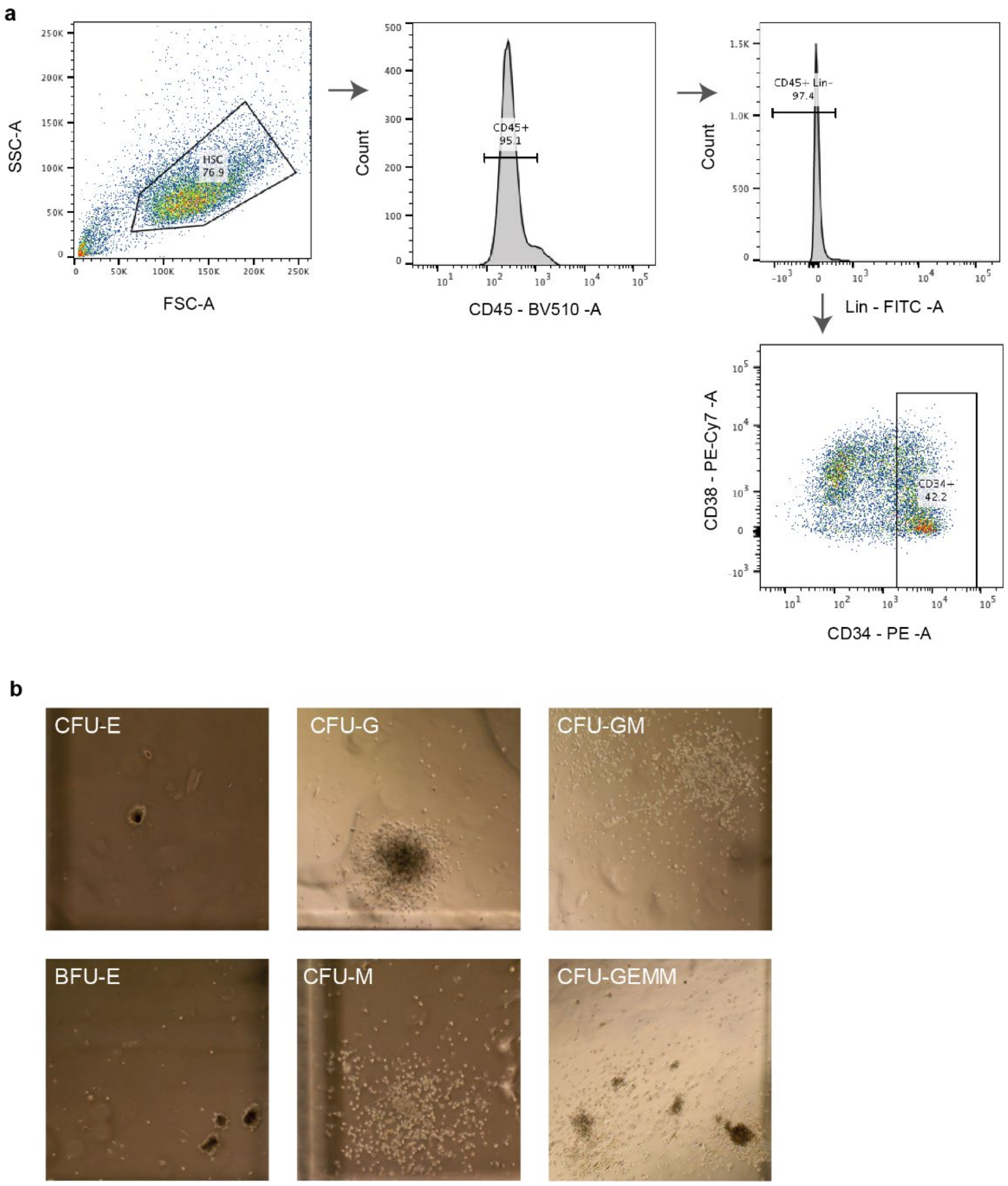

Supplementary Figure 11 | LTC-IC FACS gating strategy and colony phenotyping. a, Sample data taken from -gel conditions, from 1 biological donor. FSC-A versus SSC-A plot used to identify viable cells; gate added on $CD45^{+ve}$ cells to remove any PerSCs; Lin^{-ve} gate used to remove any committed progenitors and final gate added on $CD34^{+ve}$ population. These cells were collected and used in the LTC-IC assay. **b**, Representative images of colony types in colony forming unit (CFU) assay after 7 days. CFU-E; contain ~8-200 haemoglobinised erythroblasts. BFU-E; clusters of erythroid progenitors with high proliferative capacity. CFU-G; homogenous population of granulocyte progenitors. CFU-M; homogenous population of macrophage progenitors. CFU-GM; heterogenous population of macrophages and granulocytes. CFU-GEMM; multilineage progenitors that give rise to erythroid, granulocyte, macrophage, and megakaryocyte lineages.

a

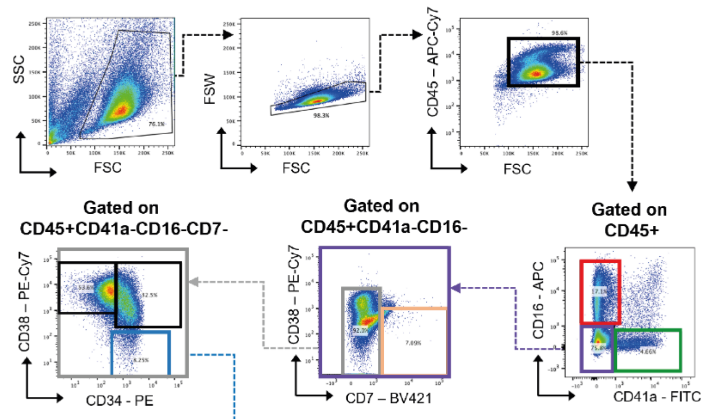

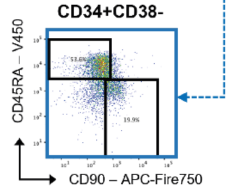

Gate	Population
CD45+	Hematopoietic
CD45+ CD41a+	Megakaryocyte
CD45+ CD16+	Monocytes, B cells, Neutrophils
CD45+ CD7+	T cells
CD45+CD41a-CD16-CD7-CD38+CD34+	Multipotent Progenitors
CD45+CD41a-CD16-CD7-CD38+CD34-	Myeloid Progenitors
CD45+CD41a-CD16-CD7-CD38-CD34+	CD34+ Progenitor Population
CD45+CD41a-CD16-CD7-CD38-CD34+CD90+CD45RA-	LT-HSC
CD45+CD41a-CD16-CD7-CD38-CD34+CD90-CD45RA+	ST-HSC

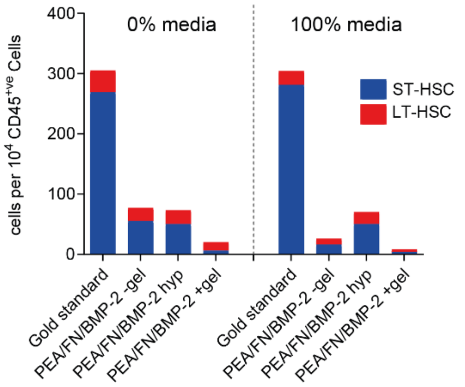

b

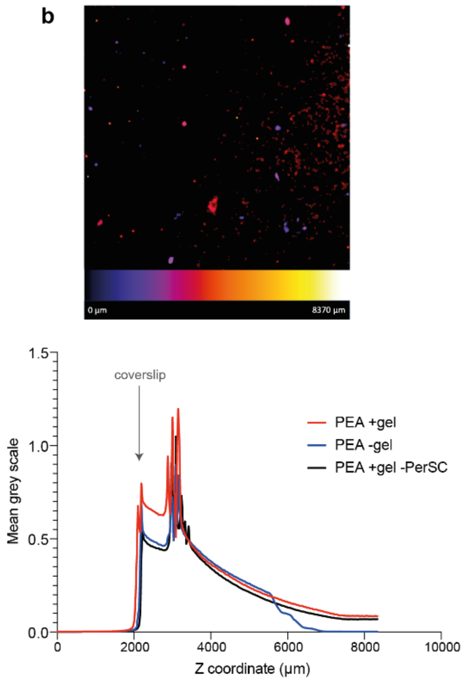

Supplementary Figure 12 | LT-HSC and ST-HSC phenotyping by flow cytometry. a, Gating strategy for extended flow cytometry panel comparing markers for LT-HSCs and ST-HSCs. Graph shows number of LT-HSC/ST-HSC per CD45+ cells and demonstrates expansion of the ST-HSC compartment in the gold standard, whereas PEA +gel maintains a population of LT-HSCs. N = 3 material replicates pooled from one biological donor. **b**, HSC migration in collagen gels was assessed via NucRed staining of HSCs and distribution in the gels measured via z-projections at day 5. The histogram shows most HSCs migrate down toward the coverslip, regardless of the presence of PerSCs in the niche models.

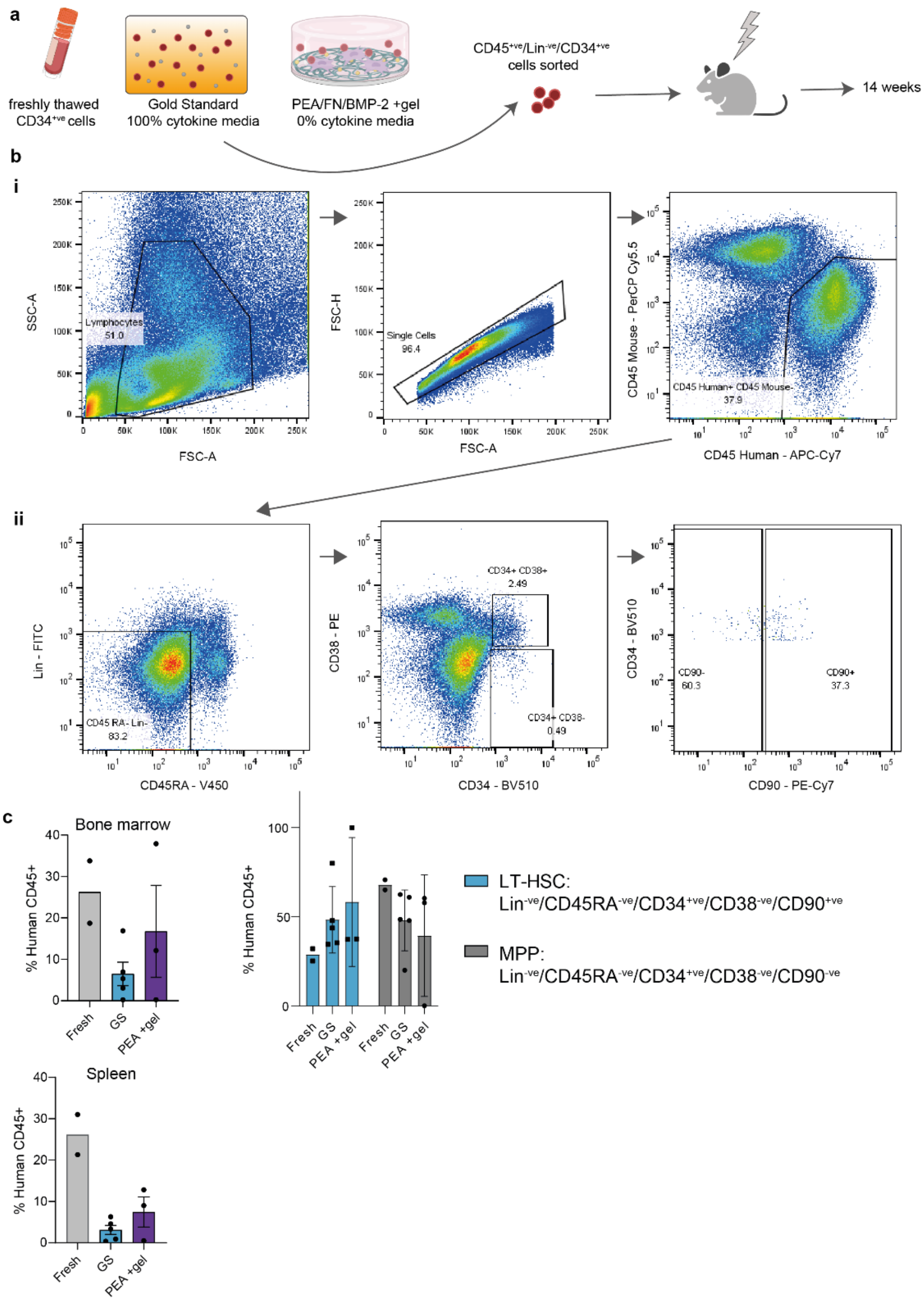

Supplementary Figure 13 | In vivo reconstitution of CD34^{+ve} cells from bioengineered niches. **a**, schematic shows experimental set up, briefly CD34^{+ve} cells were seeded in gold standard (GS) media with 100% cytokines, or in PEA/FN/BMP-2 +gel niches in 0% cytokine media. After 5 days CD45^{+ve}/Lin^{-ve}/CD34^{+ve} cells were sorted and transplanted into NOD-RAG-gc$^{-/-}$ mice, to establish the lineage potential of human HSCs derived from indicated culture conditions in vivo. b, Representative gating strategy i. for analysis of % human CD45+ve cells in peripheral blood, and ii. for stem cells marker analysis. c, Percentage human CD45+ve cells in the BM and spleen, with comparison of % LT-HSCs and multipotent progenitors (MPPs) within the CD45+ve population of the BM. Number of recipient mice; fresh (positive control) = 2, PEA +gel = 3, gold standard = 5. A, created with BioRender.com released under a Creative Commons Attribution-NonCommercial-NoDerivs 4.0 International license (https://creativecommons.org/licenses/by-nc-nd/4.0/deed.en).

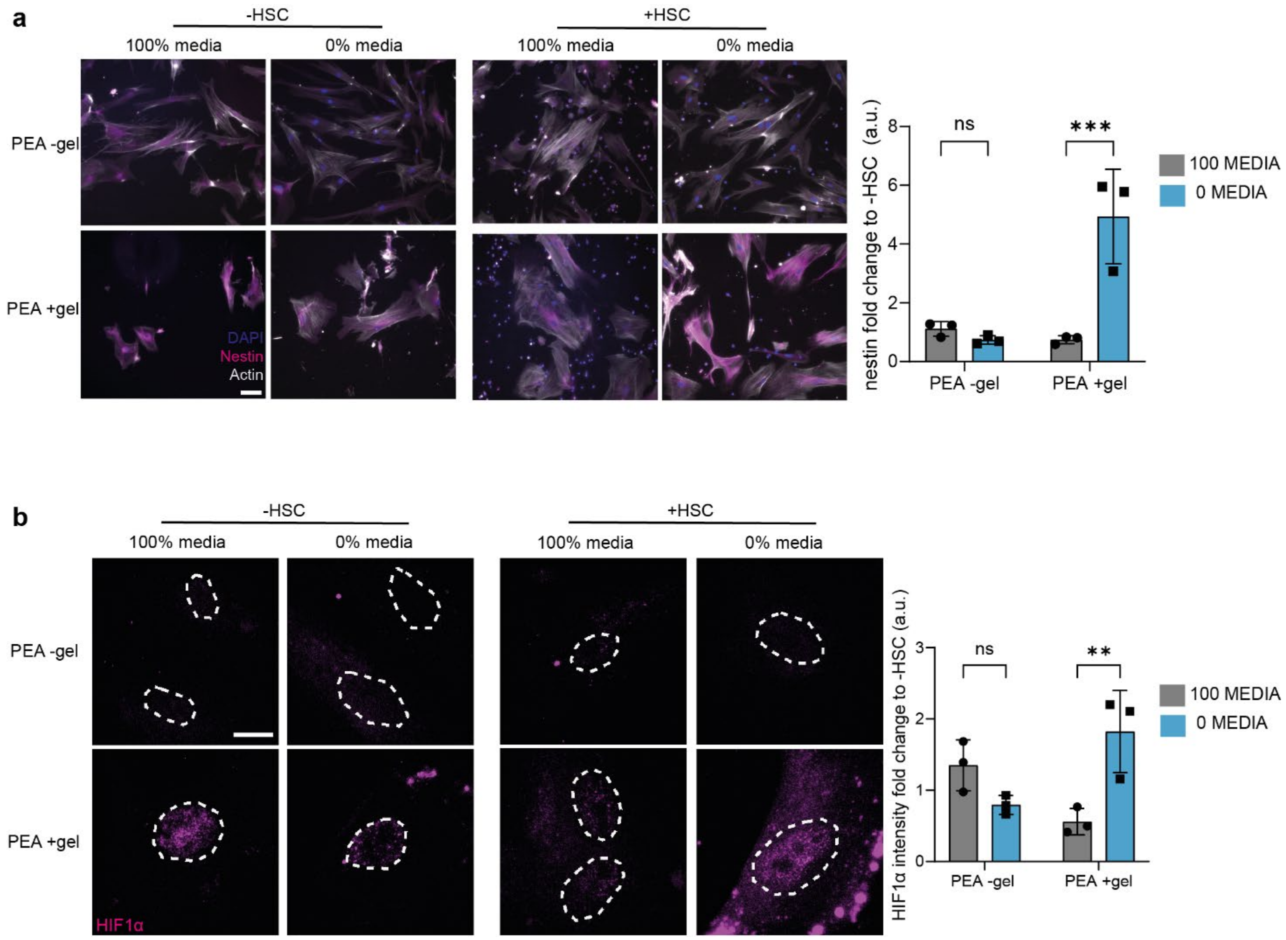

Supplementary Figure 14 | PerSC phenotype after HSC co-culture at day 19. ***a***, *Nestin and* ***b***, *HIF1a co-localisation to the nucleus are both significantly increased in PerSCs during HSC co-culture in PEA/FN/BMP-2 +gel (PEA +gel) niches, only when 0% cytokine media is used (the LT-HSC supportive microenvironment).* ***a***, *scale bar = 100 µm, magenta = nestin, grey = actin, blue = DAPI.* ***b***, *scale bar = 100 µm, dashed white line represents nuclear mask detected by DAPI staining, magenta = HIF1a. n = 3 material replicates from 1 biological donor. Actin/DAPI outlines were used to measure individual cell/nuclei nestin/ HIF1a integrated intensity with background correction. Statistics: **= p<0.005, ***= p<0.001 determined by two-way ANOVA followed by Bonferroni's multiple comparison test.*

a

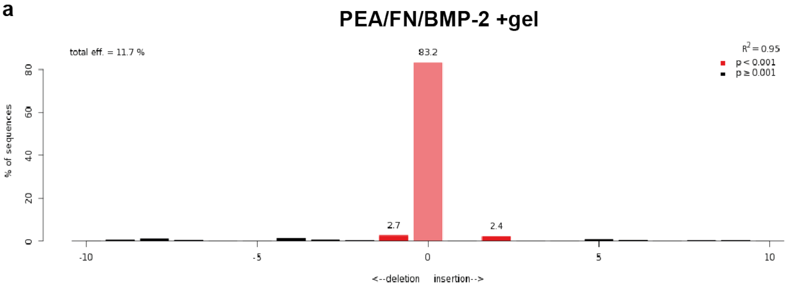

b

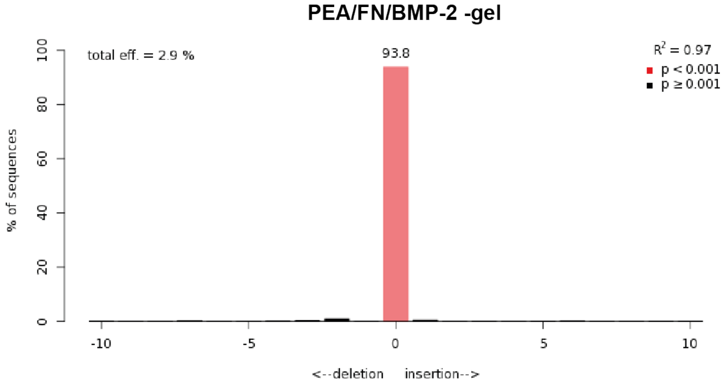

Supplementary Figure 15 | TIDE analysis of CRISPR-edited HSCs after co-culture in niches. CD34^{+ve} cells were edited using CRISPR targeting the AAVS1 locus and co-cultured in control gold standard media, or in niche models, for 5 days post-editing. Culture in PEA+gel niches enhanced editing, **a** & **b**, shows indel spectrum for PEA+gel and PEA-gel HSCs cultured in 0% media, respectively.

Supplementary Table 1| Differentiation-related genes of interest. TGF, transforming growth factor; MSC, mesenchymal stem cell; BMP, bone morphogenetic protein, SMAD, mothers against decapentaplegic; ERK, extracellular related kinase.

**Gene**	**Definition**	**Summary**	**Supp refs**
BDNF	Brain-Derived Neurotrophic Factor	Neurogenesis modulator.	[1,2]
BMP2	Bone Morphogenetic Protein-2	TGF-β superfamily member signalling molecules, involved in bone formation.	[3–5]
RUNX2	Runt-Related Transcription Factor-2	Transcriptional master regulator of osteogenesis in MSCs.	[6–8]
SMAD4	SMAD Family Member 4	Involved in canonical BMP signalling.	[9]
BMP6	Bone Morphogenetic Protein-6	TGF-β superfamily member signalling molecules, expressed in hypertrophic cartilage.	[5,10]
GFAP	Glial Fibrillary Acidic Protein	Intermediate filament protein found in astroglia cytoskeleton.	[11,12]
BGLAP	Osteocalcin	Expressed and secreted by osteoblasts.	[8,13–15]
BMP4	Bone Morphogenetic Protein 4	TGF-β superfamily member, role in embryogenesis and osteogenesis.	[5,16,17]
ALPP	Alkaline Phosphatase	Metalloenzyme, catalyses hydrolysis of phosphoric acid monoesters. Involved in bone formation.	[7,8]
SP7	Osterix	Bone-specific transcription factor. Required for osteoblast differentiation.	[18]
ADIPOQ	Adiponectin	Adipocyte-specific secretory protein.	[19–21]
TGFB3	Transforming Growth Factor Beta 3	Bind TGF-β receptors and activate SMAD signalling.	[9,22]
EGF	Epidermal Growth Factor	Role in growth, proliferation and differentiation of several cell types.	[23]
VWF	Von Willebrand Factor	Haemostatic plasma glycoprotein, expressed by endothelial cells and some HSCs.	[22,24,25]
SPARC	Osteonectin	Matrix-associated protein required for collagen calcification in bone.	[26]
BMPR1A	Bone Morphogenetic Protein Receptor Type 1A	Transmembrane serine/threonine kinase receptors for TGF-β superfamily ligands. Involved in bone formation.	[7,27]
ALPL	Alkaline Phosphatase, Biomineralization Associated	Metalloenzyme, catalyses hydrolysis of phosphoric acid monoesters. Involved in bone formation.	[7,8]

PPARG	Peroxisome Proliferator Activated Receptor Gamma	Master regulator of adipocyte differentiation.	[28]
BMPR2	Bone Morphogenetic Protein Receptor Type 2	Transmembrane serine/threonine kinase receptors for TGF-β superfamily ligands. Involved in SMAD signalling.	[29,30]
COL1A1	Collagen Type I Alpha Chain	Pro-alpha chains of type I collagen.	[7]
SOX9	SRY-Box Transcription Factor 9	Master regulator of chondrogenic differentiation in MSCs.	[31]
BMP7	Bone Morphogenetic Protein 7	TGF-β superfamily member signalling molecule, promotes osteoblast differentiation.	[32,33]
FGF10	Fibroblast Growth Factor 10	Role in cell division, proliferation, differentiation and survival. Important in development.	[34]
BMPR1B	Bone Morphogenetic Protein Receptor Type 1B	Transmembrane serine/threonine kinase receptors for TGF-β superfamily ligands. Involved in SMAD signalling.	[35]
SPP1	Osteopontin	Non-collagenous protein present in bone matrix.	[8,36,37]
FGF2	Fibroblast Growth Factor 2	Role in cell division, proliferation, differentiation and survival. Can signal through ERK to activate RUNX2.	[38]

Supplementary Table 2 | Niche-related genes of interest. BM, bone marrow; HSC, hematopoietic stem cell; MSC, mesenchymal stem cell; ECM, extracellular matrix; VEGF, vascular endothelial growth factor; ESC, embryonic stem cell.

**Gene**	**Definition**	**Summary**	**Supp refs**
CDH2	Cadherin 2	Expressed by osteolineage cells in the BM niche.	[39–43]
ALCAM	Activated Leukocyte Cell Adhesion Molecule	MSC/pericyte marker.	[24,44]
ITGAV	Integrin Subunit Alpha V	Expressed by nestin+ perivascular stromal cells in the BM niche.	[44]
CSPG4	Chondroitin Sulphate Proteoglycan 4 (NG2; Nerve/Glial antigen 2)	Expressed by arteriole lining pericytes in the BM niche.	[45–47]
JAG1	Jagged Canonical Notch Ligand 1	Expressed by BM stromal cells and osteoblasts. May activate Notch signalling in HSCs.	[47,48]
KITLG	KIT Ligand	Stem Cell Factor (SCF); HSC maintenance growth factor produced by Lepr+ and Nestin+ stromal and endothelial cells in the niche.	[49]
LIF	Leukaemia Inhibitory Factor	Interleukin-6 family cytokine, important in haematopoietic differentiation and regulation.	[50,51]
ITGB1	Integrin Subunit Beta 1	Expressed by HSCs and MSCs in the BM niche.	[4,52,53]
PDGFRA	Platelet Derived Growth Factor Receptor Alpha	Expressed by a subset of CD51+ stromal cells in the BM niche that correspond to increased nestin expression.	[44]
MMP2	Matrix Metallopeptidase 2	Shown to release CXCL12 from the ECM.	[54–56]
ICAM1	Intercellular Adhesion Molecule 1	Expressed by immunomodulatory MSCs and by sinusoidal endothelial cells in the BM niche.	[22,57]
CXCL12	C-X-C Motif Chemokine Ligand 12	Stromal Cell-Derived Factor 1(SDF-1). HSC regulatory cytokine produced by nestin+ perivascular cells in the BM niche.	[46]
ITGAX	Integrin Subunit Alpha X	Expressed in the BM niche by lymphocytes.	[58]
PDGFRB	Platelet Derived Growth Factor Receptor Beta	Expressed by endothelial cells lining BM arterioles and sinusoids.	[49]
LEPR	Leptin Receptor	Expressed by sinusoid-associated cells in the bone marrow niche.	[45–47]
VCAM1	Vascular Cell Adhesion Molecule 1	Involved in blood cell retention in the BM niche, ligands is integrinβ1.	[46,49]
NOTCH1	Notch Receptor 1	Roles in osteoblast and hematopoietic cell differentiation and maturation.	[59–61]

MCAM	Melanoma Cell Adhesion Molecule (CD146)	Marker for pericytes and stromal stem cells.	[24,44]
NCAM1	Neural Cell Adhesion Molecule 1	Considered an early neural progenitor marker – correlated with nestin expression.	[62]
NES	Nestin	Nestin+ pericytes and stromal cells regulate HSC activity in the BM niche.	[44,45,47,63–65]
THY1	Thy-1 Cell Surface Antigen	CD90; Involved in early BM niche development, used as MSC/HSC marker.	[66–68]
CSF2	Colony Stimulating Factor 2	Granulocyte macrophage-colony stimulating factor (GM-CSF). Produced in multiple cell types the BM niche.	[69,70]
KDR	Kinase Insert Domain Receptor	VEGF receptor. MSC marker.	[71,72]
THPO	Thrombopoietin	Osteoblast-derived HSC maintenance cytokine.	[73]
NGFR	Nerve Growth Factor Receptor	MSC marker.	[74]
VIM	Vimentin	Intermediate filament protein expressed in mesenchymal cells. Polymerisation partner of nestin.	[75]
POU5F1	Octamer-Binding Protein 4	Regulates self-renewal and differentiation in MSCs. Shown to be regulated by hypoxia in cultured ESCs.	[76,77]
VEGFA	Vascular Endothelial Growth Factor A	Regulator of blood vessel formation. Required for HSC survival. Produced in hypoxia.	[78]

Supplementary Table 3| Flow cytometry antibodies for PerSC phenotyping.

Marker	Fluorophore	Clone	Supplier	Catalogue number
Lepr	APC (panel 1)	REA361	Miltenyi Biotech	130-105-211
CD51	APC (panel 2)	REA181	Miltenyi Biotech	130-100-552
CD90	APC-Cy7 (panel 1)	REA897	Miltenyi Biotech	130-114-905
CD31	APC-Cy7 (panel 2)	REA730	Miltenyi Biotech	130-110-810
CD29	FITC (panel 1)	TS2/16	ThermoFisher, eBioscience	11-0299-42
NG2	PE (panel 1)	1E6.4	Miltenyi Biotech	130-100-468
CD140a	PE (panel 2)	16A1	Biolegend	323505
CD146	PerCP-Cy5.5 (panel 1)	P1H12	Biolegend	361009
CD166	PerCP-eFluor710 (panel 2)	3A6	ThermoFisher, eBioscience	46-1668-42
CD140b	PE-Cy7 (panel 2)	REA363	Miltenyi Biotech	130-105-323
CD105	eFluor 450 (panel 2)	43A4E1	Miltenyi Biotech	130-099-667

Supplementary Table 4| Flow cytometry antibodies for HSC phenotyping, LTC-IC and in vivo assay sorting.

Marker	Fluorophore	Clone	Supplier	Catalogue number
CD34	PE	4H11	ThermoFisher, eBioscience	12-0349-42
CD45	APC-Cy7	2D1	BD Biosciences	561863
CD38	PE-Cy7	HB7	ThermoFisher, eBioscience	25-0388-42
Lineage cocktail	FITC	-	ThermoFisher, eBioscience	22-7778-72
CD16	APC	3G8	ThermoFisher, eBioscience	47-0166-42
CD7	BV421	M-T701	BD Biosciences	562635
CD90	APC-Fire750	5E10	Biolegend	328138
CD45RA	V450	HI100	ThermoFisher, eBioscience	14-0458-82
CD41a	FITC	HIP8	ThermoFisher, eBioscience	11-0419-42
CD45	BV510	2D1	Biolegend	368526
CD90	PerCP-Cy5.5	5E10	ThermoFisher, eBioscience	45-0909-42
CD45RA	APC-Cy7	HI100	ThermoFisher, eBioscience	47-0458-42

Supplementary Table 5 | Flow cytometry antibodies for HSC phenotyping for in vivo experiments.

Marker	Fluorophore	Clone	Supplier	Catalogue number
Lin	FITC	-	ThermoFisher, eBioscience	22-7778-72
CD34	BV510	581	Biolegend	343528
CD45 (human)	APC-CY7	2D1	BD Biosciences	557833
CD45 (Mouse)	PerCP Cy5.5	30-F11	BD Biosciences	561869
CD38	PE	HIT2	BD Biosciences	555460
CD90	PE-Cy7	5E10	BD Biosciences	561558
CD123	PE Cy7	7G3	BD Biosciences	560826
CD11b	Pacific blue	ICRF44	BD Biosciences	558123
CD8	BV510	SK1	BD Biosciences	563919
CD3	PE	17A2	BD Biosciences	100205

CD45RA	Efluor 450	HI100	Thermofisher	48-0458-42
CD56	PE-Cy7	B159	BD Biosciences	557747
CD19	APC	HIB19	BD Biosciences	555415
FC Block (CD16/32)	-	2.4G2	BD Biosciences	569324
UltraComp eBeads	-	-	Themofisher	01-2222-42

Supplementary references

1. Reyes, J. H. *et al.* Glutamatergic neuronal differentiation of mouse embryonic stem cells after transient expression of neurogenin 1 and treatment with BDNF and GDNF: In vitro and in vivo studies. *J. Neurosci.* **28**, 12622–12631 (2008).

2. Fuentealba, L. C., Obernier, K. & Alvarez-Buylla, A. Adult neural stem cells bridge their niche. *Cell Stem Cell* **10**, 698–708 (2012).

3. Dobre, O. *et al.* A Hydrogel Platform that Incorporates Laminin Isoforms for Efficient Presentation of Growth Factors – Neural Growth and Osteogenesis. *Adv. Funct. Mater.* **31**, (2021).

4. Martino, M. M. *et al.* Engineering the growth factor microenvironment with fibronectin domains to promote wound and bone tissue healing. *Sci. Transl. Med.* **3**, 100ra89 (2011).

5. Chen, D., Zhao, M. & Mundy, G. R. Bone morphogenetic proteins. *Growth Factors* **22**, 233–241 (2004).

6. Bruderer, M., Richards, R. G., Alini, M. & Stoddart, M. J. Role and regulation of runx2 in osteogenesis. *Eur. Cells Mater.* **28**, 269–286 (2014).

7. Long, F. Building strong bones: Molecular regulation of the osteoblast lineage. *Nat. Rev. Mol. Cell Biol.* **13**, 27–38 (2012).

8. Yang, J. *et al.* Nanotopographical Induction of Osteogenesis through Adhesion, Bone Morphogenic Protein Cosignaling, and Regulation of MicroRNAs. *ACS Nano* **8**, 9941–9953 (2014).

9. Heldin, C.-H., Kohei, M. & Peter, D. TFG-beta signalling from cell membrane to nucleas through SMAD proteins. *Nature* **390**, 465–71 (1997).

10. Kugimiya, F. *et al.* Involvement of endogenous bone morphogenetic protein (BMP) 2 and BMP6 in bone formation. *J. Biol. Chem.* **280**, 35704–35712 (2005).

11. Hol, E. M. & Pekny, M. Glial fibrillary acidic protein (GFAP) and the astrocyte intermediate filament system in diseases of the central nervous system. *Curr. Opin. Cell Biol.* **32**, 121–130 (2015).

12. Yamazaki, S. *et al.* Nonmyelinating schwann cells maintain hematopoietic stem cell hibernation in the bone marrow niche. *Cell* **147**, 1146–1158 (2011).

13. Moser, S. C. & van der Eerden, B. C. J. Osteocalcin — A versatile bone-derived hormone. *Front. Endocrinol. (Lausanne).* **10**, 4–9 (2019).

14. Dalby, M. J., Gadegaard, N. & Oreffo, R. O. C. Harnessing nanotopography and integrin-matrix

interactions to influence stem cell fate. *Nat. Mater.* **13**, 558–569 (2014).

15. Curran, J. M., Chen, R. & Hunt, J. A. The guidance of human mesenchymal stem cell differentiation in vitro by controlled modifications to the cell substrate. *Biomaterials* **27**, 4783–4793 (2006).

16. Goldman, D. C. *et al.* BMP4 regulates the hematopoietic stem cell niche. *Blood* **114**, 4393–4401 (2009).

17. Re'Em, T., Witte, F., Willbold, E., Ruvinov, E. & Cohen, S. Simultaneous regeneration of articular cartilage and subchondral bone induced by spatially presented TGF-beta and BMP-4 in a bilayer affinity binding system. *Acta Biomater.* **8**, 3283–3293 (2012).

18. Nakashima, K. *et al.* The Novel Zinc Finger-Containing Transcription Factor Osterix Is Required for Osteoblast Differentiation and Bone Formation. *Cell* **108**, 17–29 (2002).

19. Yokota, T. *et al.* Adiponectin, a new member of the family of soluble defense collagens, negatively regulates the growth of myelomonocytic progenitors and the functions of macrophages. *Blood* **96**, 1723–32 (2000).

20. Maeda, K. *et al.* cDNA Cloning and Expression of a Novel Adipose Specific Collagen-like Factor, apM1 (dioseost Abundant Gene Transcript 1). *Biochem. Biophys. Res. Commun.* **221**, 286–289 (1996).

21. Saraiva, L. R. *et al.* Adipocyte Accumulation in the Bone Marrow during Obesity and Aging Impairs Stem Cell-Based Hematopoietic and Bone Regeneration. *Cell Stem Cell* **20**, 771-784.e6 (2017).

22. Xu, C. *et al.* Stem cell factor is selectively secreted by arterial endothelial cells in bone marrow. *Nat. Commun.* **9**, 1–13 (2018).

23. Krampera, M. *et al.* HB-EGF/HER-1 signaling in bone marrow mesenchymal stem cells: Inducing cell expansion and reversibly preventing multilineage differentiation. *Blood* **106**, 59–66 (2005).

24. Crisan, M. *et al.* A Perivascular Origin for Mesenchymal Stem Cells in Multiple Human Organs. *Cell Stem Cell* **3**, 301–313 (2008).

25. Pinho, S. *et al.* Lineage-Biased Hematopoietic Stem Cells Are Regulated by Distinct Niches. *Dev. Cell* **44**, 634-641.e4 (2018).

26. Termine, J. D. *et al.* Osteonectin, a bone-specific protein linking mineral to collagen. *Cell* **26**, 99–105 (1981).

27. Cheng, Z. A. *et al.* Nanoscale Coatings for Ultralow Dose BMP-2-Driven Regeneration of Critical-Sized Bone Defects. *Adv. Sci.* **1800361**, 1800361 (2018).

28. Lehrke, M. & Lazar, M. A. The many faces of PPARγ. *Cell* **123**, 993–999 (2005).

29. Lowery, J. W. *et al.* Loss of BMPR2 leads to high bone mass due to increased osteoblast activity. *J. Cell Sci.* **128**, 1308–1315 (2015).

30. Lavery, K., Swain, P., Falb, D. & Alaoui-Ismaili, M. H. BMP-2/4 and BMP-6/7 differentially utilize cell surface receptors to induce osteoblastic differentiation of human bone marrow-derived mesenchymal stem cells. *J. Biol. Chem.* **283**, 20948–20958 (2008).

31. Bi, W., Deng, J. M., Zhang, Z., Behringer, R. R. & De Crombrugghe, B. Sox9 is required for cartilage formation. *Nat. Genet.* **22**, 85–89 (1999).

32. Piccolo, S., Sasai, Y., Lu, B. & De Robertis, E. M. Dorsoventral patterning in Xenopus: Inhibition of ventral signals by direct binding of chordin to BMP-4. *Cell* **86**, 589–598 (1996).

33. Ducy, P., Zhang, R., Geoffroy, V., Ridall, A. L. & Karsenty, G. Osf2/Cbfa1: A transcriptional activator of osteoblast differentiation. *Cell* **89**, 747–754 (1997).

34. Watson, J. & Francavilla, C. Regulation of FGF10 Signaling in Development and Disease. *Front. Genet.* **9**, 1–10 (2018).

35. Shi, C. *et al.* Deletion of BMP receptor type IB decreased bone mass in association with compromised osteoblastic differentiation of bone marrow mesenchymal progenitors. *Sci. Rep.* **6**, 1–13 (2016).

36. Noda, M. & Denhardt, D. T. Chapter 18 - Osteopontin. *Princ. Bone Biol.* 351–366 (2008).

37. Mark, M. P. *et al.* Immunohistochemical Phosphoprotein Demonstration of a 44-lcD in Developing Rat Bones1 that picture. *J. Histochem. Cytochem* (1987).

38. Park, O., Kim, H., Woo, K., Baek, J. & Ryoo, H. FGF2-activated ERK Mitogen-activated Protein Kinase Enhances Runx2 Acetylation and Stabilization *. **285**, 3568–3574 (2010).

39. Zhang, J. *et al.* Identification of the haematopoietic stem cell niche and control of the niche size. *Nature* **425**, 836–841 (2003).

40. Haug, J. S. *et al.* Article N-Cadherin Expression Level Distinguishes Reserved versus Primed States of Hematopoietic Stem Cells. 367–379 (2008) doi:10.1016/j.stem.2008.01.017.

41. Hosokawa, K. *et al.* Cadherin-Based Adhesion Is a Potential Target for Niche Manipulation to Protect Hematopoietic Stem Cells in Adult Bone Marrow. *Stem Cell* **6**, 194–198 (2010).

42. Kiel, M. J., Acar, M., Radice, G. L. & Morrison, S. J. Hematopoietic Stem Cells Do Not Depend on N-Cadherin to Regulate Their Maintenance. *Cell Stem Cell* **4**, 170–179 (2009).

43. Greenbaum, A. M., Revollo, L. D., Woloszynek, J. R., Civitelli, R. & Link, D. C. N-cadherin in osteolineage cells is not required for maintenance of hematopoietic stem cells. *Blood* **120**, 295–302 (2012).

44. Pinho, S. *et al.* PDGFR A and CD51 mark human stem cells capable of hematopoietic progenitor cell expansion. *J. Exp. Med.* **210**, 1351–1367 (2013).

45. Kunisaki, Y. *et al.* Arteriolar niches maintain haematopoietic stem cell quiescence. *Nature* **502**, 637–643 (2013).

46. Asada, N. *et al.* Differential cytokine contributions of perivascular haematopoietic stem cell niches. (2017) doi:10.1038/ncb3475.

47. Baccin, C. *et al.* Combined single-cell and spatial transcriptomics reveal the molecular, cellular and spatial bone marrow niche organization. *Nat. Cell Biol.* **22**, 38–48 (2020).

48. Calvi, L. M. *et al.* Osteoblastic cells regulate the haematopoietic stem cell niche. *Nature* **425**, 841–846 (2003).

49. Ding, L., Saunders, T. L., Enikolopov, G. & Morrison, S. J. Endothelial and perivascular cells

maintain haematopoietic stem cells. *Nature* **481**, 457–62 (2012).

50. Escary, J., Perreau, J., Dumenilt, D., Ezine, S. & Brulet, P. Leukaemia inhibitory factor is necessary for maintenance of haematopoietic stem cells and thymocyte stimulation. **363**, 361–364 (1993).

51. Ranga, A. *et al.* 3D niche microarrays for systems-level analyses of cell fate. *Nat. Commun.* **5**, 1–10 (2014).

52. Hadland, B. *et al.* Engineering a niche supporting hematopoietic stem cell development using integrated single-cell transcriptomics. *Nat. Commun.* **13**, 1–17 (2022).

53. Williams, D. A., Rios, M., Stephens, C. & Patel, V. P. Fibronectin and VLA-4 in haematopoietic stem cell-microenvironment interactions. *Nature* **352**, 438–441 (1991).

54. Nyman, J. S. *et al.* Differential Effects Between the Loss of MMP-2 and MMP-9 on Structural and Tissue-Level Properties of Bone. **26**, 1252–1260 (2011).

55. Clutter, S. D., Fortney, J. & Gibson, L. F. MMP-2 is required for bone marrow stromal cell support of pro – B-cell chemotaxis. **33**, 1192–1200 (2005).

56. Saw, S., Weiss, A., Khokha, R. & Waterhouse, P. D. Metalloproteases : On the Watch in the Hematopoietic Niche. *Trends Immunol.* **40**, 1053–1070 (2019).

57. Ghannam, S., Pène, J., Torcy-moquet, G., Jorgensen, C. & Yssel, H. Mesenchymal Stem Cells Inhibit Human Th17 Cell Differentiation and Function and Induce a T Regulatory Cell Phenotype. (2022) doi:10.4049/jimmunol.0902007.

58. Villani, A. C. *et al.* Single-cell RNA-seq reveals new types of human blood dendritic cells, monocytes, and progenitors. *Science (80-.).* **356**, (2017).

59. Maillard, I. *et al.* Article Canonical Notch Signaling Is Dispensable for the Maintenance of Adult Hematopoietic Stem Cells. **1**, (2006).

60. Butler, J. M. *et al.* Endothelial Cells Are Essential for the Self-Renewal and Repopulation of Notch-Dependent Hematopoietic Stem Cells. *Cell Stem Cell* **6**, 251–264 (2010).

61. Mancini, S. J. C. *et al.* Jagged1-dependent Notch signaling is dispensable for hematopoietic stem cell self-renewal and differentiation. *Blood* **105**, 2340–2342 (2005).

62. Engler, A. J., Sen, S., Sweeney, H. L. & Discher, D. E. Matrix elasticity directs stem cell lineage specification. *Cell* **126**, 677–89 (2006).

63. Ding, L. & Morrison, S. J. Haematopoietic stem cells and early lymphoid progenitors occupy distinct bone marrow niches. *Nature* **495**, 231–235 (2013).

64. Ding, L., Saunders, T. L., Enikolopov, G. & Morrison, S. J. Endothelial and perivascular cells maintain haematopoietic stem cells. *Nature* **481**, 457–62 (2012).

65. Méndez-Ferrer, S. *et al.* Mesenchymal and haematopoietic stem cells form a unique bone marrow niche. *Nature* **466**, 829–834 (2010).

66. Chan, C. K. F. *et al.* Endochondral ossification is required for haematopoietic stem-cell niche formation. *Nature* **457**, 490–494 (2009).

67. Notta, F. *et al.* Isolation of Single Human Hematopoietic. *Science (80-.).* **333**, 218–222 (2011).

68. Maleki, M., Ghanbarvand, F. & Behvarz, M. R. Comparison of Mesenchymal Stem Cell Markers in Multiple Human Adult Stem Cells. **7**, 118–126 (2014).

69. Taichman, R., Reilly, M. J. & Emerson, S. G. Human osteoblasts support human hematopoietic progenitor cells in vitro bone marrow cultures. *Blood* **87**, 518–524 (1996).

70. Trapnell, B. C. & Abe, S. Colony stimulating factors. 540–546 (2004).

71. Cortés, F., Debacker, C., Péault, B. & Labastie, M. C. Differential expression of KDR/VEGFR-2 and CD34 during mesoderm development of the early human embryo. *Mech. Dev.* **83**, 161–164 (1999).

72. Blaber, E. A. *et al.* Mechanical unloading of bone in microgravity reduces mesenchymal and hematopoietic stem cell-mediated tissue regeneration. *Stem Cell Res.* **13**, 181–201 (2014).

73. Yoshihara, H. *et al.* Thrombopoietin/MPL Signaling Regulates Hematopoietic Stem Cell Quiescence and Interaction with the Osteoblastic Niche. *Cell Stem Cell* **1**, 685–697 (2007).

74. Kumar, A. *et al.* Specification and Diversification of Pericytes and Smooth Muscle Cells from Mesenchymoangioblasts. *Cell Rep.* **19**, 1902–1916 (2017).

75. Johnson, C. P., Tang, H., Carag, C., Speicher, D. W. & Discher, D. E. Forced Unfolding of Proteins Within Cells. *Science (80-.).* **317**, 663–667 (2007).

76. Forristal, C. E., Wright, K. L., Hanley, N. A., Oreffo, R. O. C. & Houghton, F. D. Hypoxia inducible factors regulate pluripotency and proliferation in human embryonic stem cells cultured at reduced oxygen tensions. *Reproduction* **139**, 85–97 (2010).

77. Tsai, C. C., Su, P. F., Huang, Y. F., Yew, T. L. & Hung, S. C. Oct4 and Nanog Directly Regulate Dnmt1 to Maintain Self-Renewal and Undifferentiated State in Mesenchymal Stem Cells. *Mol. Cell* **47**, 169–182 (2012).

78. Gerber, H., Malik, A. K., Solar, G. P. & Sherman, D. VEGF regulates haematopoietic stem cell survival by an internal autocrine loop mechanism. *Nature* **417**, 6–10 (2002).
