# Supplementary material for: Bioengineered niches that recreate physiological extracellular matrix organisation to support long-term haematopoietic stem cells
Source: Nat Commun. 2024 Jul 10;15:5791. doi: 10.1038/s41467-024-50054-0 (PMC11237034; doi:10.1038/s41467-024-50054-0)
Supplement: Supplementary file 3 — Reporting summary [file 41467_2024_50054_MOESM3_ESM.pdf]

nature portfolio

Corresponding author(s): Matthew J Dalby

Last updated by author(s): May 3, 2024

Reporting Summary

Nature Portfolio wishes to improve the reproducibility of the work that we publish. This form provides structure for consistency and transparency in reporting. For further information on Nature Portfolio policies, see our Editorial Policies and the Editorial Policy Checklist.

Statistics

For all statistical analyses, confirm that the following items are present in the figure legend, table legend, main text, or Methods section.

n/a	Confirmed	
☐	☒	The exact sample size (*n*) for each experimental group/condition, given as a discrete number and unit of measurement
☐	☒	A statement on whether measurements were taken from distinct samples or whether the same sample was measured repeatedly
☐	☒	The statistical test(s) used AND whether they are one- or two-sided<br>*Only common tests should be described solely by name; describe more complex techniques in the Methods section.*
☐	☒	A description of all covariates tested
☐	☒	A description of any assumptions or corrections, such as tests of normality and adjustment for multiple comparisons
☐	☒	A full description of the statistical parameters including central tendency (e.g. means) or other basic estimates (e.g. regression coefficient) AND variation (e.g. standard deviation) or associated estimates of uncertainty (e.g. confidence intervals)
☐	☒	For null hypothesis testing, the test statistic (e.g. *F*, *t*, *r*) with confidence intervals, effect sizes, degrees of freedom and *P* value noted<br>*Give P values as exact values whenever suitable.*
☒	☐	For Bayesian analysis, information on the choice of priors and Markov chain Monte Carlo settings
☐	☒	For hierarchical and complex designs, identification of the appropriate level for tests and full reporting of outcomes
☒	☐	Estimates of effect sizes (e.g. Cohen's *d*, Pearson's *r*), indicating how they were calculated

Our web collection on statistics for biologists contains articles on many of the points above.

Software and code

Policy information about availability of computer code

Data collection	Invitrogen™ EVOS™ M7000 Imaging system hj7; FACSDiva software v8.0; LI-COR Image Studio™ v5.2, JPK Nanowizard 4 (JPK Instruments); Zeiss Airyscan 2 LSM 980 microscope with ZEN 3.5 blue edition software; Tecan infinite 200Pro; Liquid chromatography/mass spectrometry analysis: UltiMate 3000 Rapid Seperation Liquid Chromatography (ThermoFisher), with a 6 150 x 4.6 mm ZIC- pHILIC column with Orbitrap Exactive; Heavy labelled glucose tracing: LC-MS platform consisted of an Accela 600 HPLC system combined with an Exactive (Orbitrap) mass spectrometer (ThermoFisher). Two complementary columns were used; the zwitterionic ZICpHILLIC column (150 mm x 4.6 mm; 3.5 µm, Merck) and the reversed phase ACE C18-AR column (150 mm x 4.6 mm; 3.5 µm Hichrom; RNAseq - Illumina NextSeq 500 platform
Data analysis	R version 4.1.2, packages: ggplot2 v3.3.6; heatmap3 v1.1.9; RColorBrewer v1.1.3; dplyr v1.0.9; readr v2.1.2; pheatmap v1.0.12; pathfindR v1.6.3; JPK data processing software v5 (AFM); Prism software v9 (GraphPad); FlowJo software v0.5.3; Metaboanalyst v4; ImageJ v1.52d; Cell profiler version 2.1.1 (protocol available in available in DOI); http://shinyapps.datacurators.nl/tide/; Tecan i-control , 1.11.1.0; Heavy labelled glucose tracing: PeakML and Mz-Match-ISO. RNAseq - Cutadapt, FastQC, Kallisto, Bioconductor package DESeq2, Cluster 3.0 and Java Treeview 3.0 software, R package Genomic Ranges from BioConductor v3.065, edgeR from BioConductor 3.066, BioTools (https://www.biotools.fr), DEseq2 BioConductor package, PathFinderR package.

For manuscripts utilizing custom algorithms or software that are central to the research but not yet described in published literature, software must be made available to editors and reviewers. We strongly encourage code deposition in a community repository (e.g. GitHub). See the Nature Portfolio guidelines for submitting code & software for further information.

Data

Policy information about availability of data

All manuscripts must include a data availability statement. This statement should provide the following information, where applicable:

- Accession codes, unique identifiers, or web links for publicly available datasets
- A description of any restrictions on data availability
- For clinical datasets or third party data, please ensure that the statement adheres to our policy

All data supporting the findings in this study are available within the article and its Supplementary Information files, can be obtained from the corresponding author or can be accessed at: http://dx.doi.org/10.5525/gla.researchdata.1326. Source data are provided with this paper.
This statement has been added to the manuscript and the doi link is now live.

Research involving human participants, their data, or biological material

Policy information about studies with human participants or human data. See also policy information about sex, gender (identity/presentation), and sexual orientation and race, ethnicity and racism.

Reporting on sex and gender	This study uses PerSCs isolated from the adipose tissue of healthy consenting patients undergoing cosmetic lipectomy procedures, or from patients undergoing breast reconstruction procedure, using deep inferior epigastric perforators (DIEP) with prior written consent; as such all donors from this procedure were female. Ethical approval for the collection of adipose tissue and subsequent research was granted by the South-East Scotland Research Ethics Committee 3 (SESREC03, reference no. 10/S1103/ 45). Human CD34+ve cells were either purchased from CalTag Medsystems or STEMCELL Technologies, or isolated from the bone marrow aspirates of patients undergoing joint replacement surgery with prior written consent. The permission to use the residual tissues was given by the Greater Glasgow and Clyde NHS Biorepository. Cells used in this study are from patients aged 20-80 years old and are a mixture of male and female donors. 50/50 male/female CD34+ve cells were purchased. Both male and female cells were used in all experiments. In line with the ethics and patient confidentiality, details of age and sex were blinded to the researchers carrying out tissue isolations and subsequent work. Due to limited donor availability via both procedures no specific age group or sex was used in this study. All NRG-3GS mice were 8 -10 wk old males, and were housed at the Beatson Research Unit (University of Glasgow, UK). Experimental protocols for working with animals were approved by the local AWERB committee and national Home Office (PD6C67A47).
Reporting on race, ethnicity, or other socially relevant groupings	n/a
Population characteristics	Cells used in this study are from patients aged 20-80 years old, and are a mixture of male and female donors.
Recruitment	Cells were isolated from waste adipose tissue taken during lipectomy or DIEP procedures, or from waste bone marrow aspirate taken during routine knee or hip replacement surgery. Consent was obtained from patients prior to the procedure, This approach has been validated by local ethics committees at the hospitals where the procedures are performed.
Ethics oversight	Ethical approval for the collection of tissue and subsequent research was granted by the South-East Scotland Research Ethics Committee 3 (SESREC03, reference no. 10/S1103/ 45). Human CD34+ve cells were isolated from the bone marrow aspirates of patients undergoing joint replacement surgery. The permission to use the residual tissues was given by the Greater Glasgow and Clyde NHS Biorepository.

Note that full information on the approval of the study protocol must also be provided in the manuscript.

Field-specific reporting

Please select the one below that is the best fit for your research. If you are not sure, read the appropriate sections before making your selection.

☒ Life sciences ☐ Behavioural & social sciences ☐ Ecological, evolutionary & environmental sciences

For a reference copy of the document with all sections, see nature.com/documents/nr-reporting-summary-flat.pdf

Life sciences study design

All studies must disclose on these points even when the disclosure is negative.

Sample size	Sample size was determined on the basis of prior experience, conditions routinely included 3 or 4 replicates to ensure reproducibility and sufficient data points for statistical analysis (Cheng et al., 2018 , Donnelly et al., 2023., Ross et al., 2023, Sweeten 2019). 3 biological donors were tested for experiments related to Figures 3c-f, 5a 6c, 7b&c. 2 CD34+ donors for experiments related to figure 7d,e,g. 1 biological donor with minimum 3 material replicates for experiments related to figures 3g&h, 4, 5b-f, 6a&b, 6d&e. For CD34+ HSC cells used for in vivo analysis in figure 7f, 10 donors were used. Were multiple replicates were pooled, this was on an a a per application basis, where larger amounts of materials are required for certain techniques (RNA-seq, flow cytometry).

Data exclusions	1 sample was removed from metabolomics analysis as as it failed to produce readable peaks. 1 sample was removed from RNA-seq analysis due to insufficient RNA quality. Quality reports and peak intensity tables for these samples can be provided.
Replication	Experiments were replicated independently 3 times with different donor cells, unless otherwise stated in the figure legends.
Randomization	Experimental conditions were assigned to culture wells without bias. Standard 24 well plates were used for cell culture experiments. Due to the nature of this research, randomization is not appropriate as assignment to categories must be exact therefore no systematic randomization was required.
Blinding	Data acquisition and analysis were non-blinded, for some analysis experimenter bias was circumvented by the use of automated data analysis pipelines (cell profiler)<br>Blinding was used for data acquisition of figure 7d&e, where 2 individuals were blinded for colony counting and phenotyping, and for fig 7f where samples were blinded to the researcher performing the xenotransplantation and the flow cytometry data collection.

Reporting for specific materials, systems and methods

We require information from authors about some types of materials, experimental systems and methods used in many studies. Here, indicate whether each material, system or method listed is relevant to your study. If you are not sure if a list item applies to your research, read the appropriate section before selecting a response.

Materials & experimental systems

n/a	Involved in the study	
☐	☒	Antibodies
☐	☒	Eukaryotic cell lines
☒	☐	Palaeontology and archaeology
☐	☒	Animals and other organisms
☒	☐	Clinical data
☒	☐	Dual use research of concern
☒	☐	Plants

Methods

n/a	Involved in the study	
☒	☐	ChIP-seq
☐	☒	Flow cytometry
☒	☐	MRI-based neuroimaging

Antibodies

Antibodies used

Immunofluorescence imaging antibodies:
anti-nestin [10C2] 1:200 (mouse monoclonal, Abcam, ab22035); anti-p(Th316)-nestin [a-4] at 1:200 (mouse monoclonal, Santa Cruz Biotechnology, sc-377538); anti-HIF1α [EP1215Y] 1:300 (rabbit monoclonal, Abcam, ab51608); anti-vimentin [SP20] 1:300 (rabbit monoclonal, Thermo Fischer, MA5-16409); anti-CXCL12 1:200 (monoclonal mouse, R & D Systems, MAB350); anti-SCF 1:200 (rabbit polyclonal, Abcam, ab64677); fluorescein isothiocyanate-conjugated streptavidin (1:50; Vector Laboratories); Anti-lactate dehydrogenase (LDH) [EP1566Y] 1:200 (rabbit monoclonal, Abcam, ab52488); LI-COR anti-rabbit secondary antibody 1:2000 (926-32211); anti-pimonidazole (1:200; Hypoxyprobe™, HP1-200kit); anti-mouse secondary (1:50, Texas Red; Vector Laboratories).

For flow cytometry all dilutions were 1:100 unless otherwise stated.
Supplementary Table 3| Flow cytometry antibodies for PerSC phenotyping.
Lepr APC (panel 1) REA361 Miltenyi Biotech 130-105-211
CD51 APC (panel 2) REA181 Miltenyi Biotech 130-100-552
CD90 APC-Cy7 (panel 1) REA897 Miltenyi Biotech 130-114-905
CD31 APC-Cy7 (panel 2) REA730 Miltenyi Biotech 130-110-810
CD29 FITC (panel 1) TS2/16 ThermoFisher, eBioscience 11-0299-42
NG2 PE (panel 1) 1E6.4 Miltenyi Biotech 130-100-468
CD140a PE (panel 2) 16A1 Biolegend 323505
CD146 PerCP-Cy5.5 (panel 1) P1H12 Biolegend 361009
CD166 PerCP-eFluor710 (panel 2) 3A6 ThermoFisher, eBioscience 46-1668-42
CD140b PE-Cy7 (panel 2) REA363 Miltenyi Biotech 130-105-323
CD105 eFluor 450 (panel 2) 43A4E1 Miltenyi Biotech 130-099-667

Supplementary Table 4| Flow cytometry antibodies for HSC phenotyping, LTC-IC and in vivo assay sorting.
CD34 PE 4H11 ThermoFisher, eBioscience 12-0349-42
CD45 APC-Cy7 2D1 BD Biosciences 561863 (1:250)
CD38 PE-Cy7 HB7 ThermoFisher, eBioscience 25-0388-42
Lineage cocktail FITC - ThermoFisher, eBioscience 22-7778-72 (1:50)
CD16 APC 3G8 ThermoFisher, eBioscience 47-0166-42
CD7 BV421 M-T701 BD Biosciences 562635
CD90 APC-Fire750 5E10 Biolegend 328138 (1:200)
CD45RA V450 HI100 ThermoFisher, eBioscience 14-0458-82
CD41a FITC HIP8 ThermoFisher, eBioscience 11-0419-42
CD45 BV510 2D1 Biolegend 368526
CD90 PerCP-Cy5.5 5E10 ThermoFisher, eBioscience 45-0909-42
CD45RA APC-Cy7 HI100 ThermoFisher, eBioscience 47-0458-42

Supplementary Table 5 | Flow cytometry antibodies for HSC phenotyping for in vivo experiments.
Lin FITC - ThermoFisher, eBioscience 22-7778-72
CD34 BV510 581 Biolegend 343528
CD45 (human) APC-CY7 2D1 BD Biosciences 557833 (1:250)
CD45 (Mouse) PerCP Cy5.5 30-F11 BD Biosciences 561869 (1:250)
CD38 PE HIT2 BD Biosciences 555460
CD90 PE-Cy7 5E10 BD Biosciences 561558
CD123 PE Cy7 7G3 BD Biosciences 560826
CD11b Pacific blue ICRF44 BD Biosciences 558123
CD8 BV510 SK1 BD Biosciences 563919
CD3 PE 17A2 BD Biosciences 100205
CD45RA Efluor 450 HI100 Thermofisher 48-0458-42
CD56 PE-Cy7 B159 BD Biosciences 557747
CD19 APC HIB19 BD Biosciences 555415
FC Block (CD16/32) - 2.4G2 BD Biosciences 569324
UltraComp eBeads - - Themofisher 01-2222-42

Validation

Validation information available on the manufacturer's website as detailed below.

anti-nestin [10C2] 1:200 (mouse monoclonal, Abcam, ab22035); https://www.abcam.com/products/primary-antibodies/nestin-antibody-10c2-neural-stem-cell-marker-ab22035.html
anti-p(Th316)-nestin [a-4] at 1:200 (mouse monoclonal, Santa Cruz Biotechnology, sc-377538); https://www.scbt.com/p/p-nestin-antibody-a-4
anti-HIF1α [EP1215Y] 1:300 (rabbit monoclonal, Abcam, ab51608); https://www.abcam.com/products/primary-antibodies/hif-1-alpha-antibody-ep1215y-ab51608.html
anti-vimentin [SP20] 1:300 (rabbit monoclonal, Thermo Fischer, MA5-16409); https://www.thermofisher.com/antibody/product/Vimentin-Antibody-clone-SP20-Monoclonal/MA5-16409
anti-CXCL12 1:200 (monoclonal mouse, R & D Systems, MAB350); Arranz et al., Nature 2014, Chow et al., 2011 JEM - https://www.rndsystems.com/products/human-mouse-cxcl12-sdf-1-antibody-79018_mab350
anti-SCF 1:200 (rabbit polyclonal, Abcam, ab64677); Zhou et al., 2017 Nature Cell Biology - https://www.abcam.com/products/primary-antibodies/scf-antibody-ab64677.html
Anti-lactate dehydrogenase (LDH) [EP1566Y] 1:200 (rabbit monoclonal, Abcam, ab52488); https://www.abcam.com/products/primary-antibodies/lactate-dehydrogenase-antibody-ep1566y-ab52488.html
anti-pimonidazole (1:200; Hypoxyprobe™, HP1-200kit); Nombela-Arrieta et al., 2013 Nature Cell Biology, Sharma et al., Haematologica 2012 - http://www.hypoxyprobe.com/knowledge-center.html
Lepr APC (panel 1) REA361 Miltenyi Biotech 130-105-211 https://www.miltenyibiotec.com/GB-en/products/cd295-lepr-antibody-anti-human-reafinity-rea361.html#conjugate=apc:size=100-tests-in-200-ul
CD51 APC (panel 2) REA181 Miltenyi Biotech 130-100-552 https://www.miltenyibiotec.com/GB-en/products/cd51-antibody-anti-human-reafinity-rea181.html#conjugate=vio-bright-v423:size=100-tests-in-200-ul
CD90 APC-Cy7 (panel 1) REA897 Miltenyi Biotech 130-114-905 https://www.miltenyibiotec.com/GB-en/products/cd90-antibody-anti-human-reafinity-rea897.html#conjugate=apc-vio-770:size=100-tests-in-200-ul
CD31 APC-Cy7 (panel 2) REA730 Miltenyi Biotech 130-110-810 https://www.miltenyibiotec.com/GB-en/products/cd31-antibody-anti-human-reafinity-rea730.html#conjugate=apc-vio-770:size=100-tests-in-200-ul
CD29 FITC (panel 1) TS2/16 ThermoFisher, eBioscience 11-0299-42 https://www.thermofisher.com/order/genome-database/dataSheetPdf?producttype=antibody&productsubtype=antibody_primary&productId=11-0299-42&version=7
NG2 PE (panel 1) 1E6.4 Miltenyi Biotech 130-100-468 https://www.miltenyibiotec.com/GB-en/products/an2-antibody-anti-human-mouse-1e6-4.html#conjugate=pe:size=100-tests-in-200-ul
CD140a PE (panel 2) 16A1 Biolegend 323505 https://d1spbj2x7qk4bg.cloudfront.net/en-gb/products/pe-anti-human-cd140a-pdgfralpha-antibody-3727?pdf=true&displayInline=true&leftRightMargin=15&topBottomMargin=15&filename=PE%20anti-human%20CD140a%20(PDGFR%CE%B1)%20Antibody.pdf&v=20240412063148
CD146 PerCP-Cy5.5 (panel 1) P1H12 Biolegend 361009 https://d1spbj2x7qk4bg.cloudfront.net/en-gb/products/percp-cyanine5-5-anti-human-cd146-antibody-9273?pdf=true&displayInline=true&leftRightMargin=15&topBottomMargin=15&filename=PerCP/Cyanine5.5%20anti-human%20CD146%20Antibody.pdf&v=20240323063043
CD166 PerCP-eFluor710 (panel 2) 3A6 ThermoFisher, eBioscience 46-1668-42 https://www.thermofisher.com/order/genome-database/dataSheetPdf?producttype=antibody&productsubtype=antibody_primary&productId=46-1668-42&version=7
CD140b PE-Cy7 (panel 2) REA363 Miltenyi Biotech 130-105-323 https://www.miltenyibiotec.com/GB-en/products/cd140b-antibody-anti-human-reafinity-rea363.html#conjugate=pe-vio-770:size=100-tests-in-200-ul
CD105 eFluor 450 (panel 2) 43A4E1 Miltenyi Biotech 130-099-667 https://www.miltenyibiotec.com/GB-en/products/cd105-antibody-anti-human-43a4e1.html#conjugate=vioblue:size=100-tests-in-1-ml
CD34 PE 4H11 ThermoFisher, eBioscience 12-0349-42 Nakahara et al., 2019 Nature Cell Biology - https://www.thermofisher.com/order/genome-database/dataSheetPdf?producttype=antibody&productsubtype=antibody_primary&productId=12-0349-42&version=7
CD45 APC-Cy7 2D1 BD Biosciences 561863 (1:250) - https://www.bdbiosciences.com/en-gb/products/reagents/flow-cytometry-reagents/research-reagents/single-color-antibodies-ruo/apc-cy-7-mouse-anti-human-cd45.561863
CD38 PE-Cy7 HB7 ThermoFisher, eBioscience 25-0388-42 https://www.thermofisher.com/order/genome-database/dataSheetPdf?producttype=antibody&productsubtype=antibody_primary&productId=25-0388-42&version=7
Lineage cocktail FITC - ThermoFisher, eBioscience 22-7778-72 (1:50) https://www.thermofisher.com/order/genome-database/dataSheetPdf?producttype=antibody&productsubtype=antibody_primary&productId=22-7778-72&version=7
CD16 APC 3G8 ThermoFisher, eBioscience 47-0166-42 https://www.thermofisher.com/order/genome-database/dataSheetPdf?producttype=antibody&productsubtype=antibody_primary&productId=47-0166-42&version=7
CD7 BV421 M-T701 BD Biosciences 562635 https://www.bdbiosciences.com/en-gb/products/reagents/flow-cytometry-reagents/research-reagents/single-color-antibodies-ruo/bv421-mouse-anti-human-cd7.562635
CD90 APC-Fire750 5E10 Biolegend 328138 (1:200) https://d1spbj2x7qk4bg.cloudfront.net/en-gb/products/apc-fire-750-anti-human-cd90-thy1-antibody-15884?pdf=true&displayInline=true&leftRightMargin=15&topBottomMargin=15&filename=APC/Fire%E2%84%

A2%20750%20anti-human%20CD90%20(Thy1)%20Antibody.pdf&v=20240410063626
CD45RA V450 HI100 ThermoFisher, eBioscience 14-0458-82 https://www.thermofisher.com/order/genome-database/dataSheetPdf?producttype=antibody&productsubtype=antibody_primary&productId=14-0458-82&version=7
CD41a FITC HIP8 ThermoFisher, eBioscience 11-0419-42
CD45 BV510 2D1 Biolegend 368526 https://d1spbj2x7qk4bg.cloudfront.net/en-gb/products/brilliant-violet-510-anti-human-cd45-antibody-14685?pdf=true&displayInline=true&leftRightMargin=15&topBottomMargin=15&filename=Brilliant%20Violet%20510%E2%84%A2%20anti-human%20CD45%20Antibody.pdf&v=20240412063148
CD90 PerCP-Cy5.5 5E10 ThermoFisher, eBioscience 45-0909-42 https://www.thermofisher.com/order/genome-database/dataSheetPdf?producttype=antibody&productsubtype=antibody_primary&productId=45-0909-42&version=7
CD45RA APC-Cy7 HI100 ThermoFisher, eBioscience 47-0458-42 https://www.thermofisher.com/order/genome-database/dataSheetPdf?producttype=antibody&productsubtype=antibody_primary&productId=47-0458-42&version=7

Antibodies for in vivo validated in previous experiments from Michie lab - Kuntz et al., Nature Medicine 2017
CD34 BV510 581 Biolegend 343528 https://d1spbj2x7qk4bg.cloudfront.net/en-gb/products/brilliant-violet-510-anti-human-cd34-antibody-8450?pdf=true&displayInline=true&leftRightMargin=15&topBottomMargin=15&filename=Brilliant%20Violet%20510%E2%84%A2%20anti-human%20CD34%20Antibody.pdf&v=20240412063148
CD45 (human) APC-CY7 2D1 BD Biosciences 557833 https://www.bdbiosciences.com/en-gb/products/reagents/flow-cytometry-reagents/research-reagents/single-color-antibodies-ruo/apc-cy-7-mouse-anti-human-cd45.557833
CD45 (Mouse) PerCP Cy5.5 30-F11 BD Biosciences 561869 https://www.bdbiosciences.com/en-gb/products/reagents/flow-cytometry-reagents/research-reagents/single-color-antibodies-ruo/percp-cy-5-5-rat-anti-mouse-cd45.561869
CD38 PE HIT2 BD Biosciences 555460 https://www.bdbiosciences.com/content/dam/bdb/products/global/reagents/flow-cytometry-reagents/research-reagents/single-color-antibodies-ruo/555xxx/5554xx/555460_base/pdf/555460.pdf
CD90 PE-Cy7 5E10 BD Biosciences 561558 https://www.bdbiosciences.com/content/dam/bdb/products/global/reagents/flow-cytometry-reagents/research-reagents/single-color-antibodies-ruo/561xxx/5615xx/561558_base/pdf/561558.pdf
CD123 PE Cy7 7G3 BD Biosciences 560826
CD11b Pacific blue ICRF44 BD Biosciences 558123 https://www.bdbiosciences.com/content/dam/bdb/products/global/reagents/flow-cytometry-reagents/research-reagents/single-color-antibodies-ruo/558xxx/5581xx/558123_base/pdf/558123.pdf
CD8 BV510 SK1 BD Biosciences 563919 https://www.bdbiosciences.com/content/dam/bdb/products/global/reagents/flow-cytometry-reagents/research-reagents/single-color-antibodies-ruo/563xxx/5639xx/563919_base/pdf/563919.pdf
CD3 PE 17A2 BD Biosciences 100205 https://www.bdbiosciences.com/content/dam/bdb/products/global/reagents/flow-cytometry-reagents/research-reagents/single-color-antibodies-ruo/555xxx/5552xx/555275_base/pdf/555275.pdf
CD45RA Efluor 450 HI100 Thermofisher 48-0458-42 https://www.thermofisher.com/order/genome-database/dataSheetPdf?producttype=antibody&productsubtype=antibody_primary&productId=48-0458-42&version=7
CD56 PE-Cy7 B159 BD Biosciences 557747 https://www.bdbiosciences.com/content/dam/bdb/products/global/reagents/flow-cytometry-reagents/research-reagents/single-color-antibodies-ruo/557xxx/5577xx/557747_base/pdf/557747.pdf
CD19 APC HIB19 BD Biosciences 555415 https://www.bdbiosciences.com/content/dam/bdb/products/global/reagents/flow-cytometry-reagents/research-reagents/single-color-antibodies-ruo/555xxx/5554xx/555415_base/pdf/555415.pdf

Eukaryotic cell lines

Policy information about cell lines and Sex and Gender in Research

Cell line source(s): All cells used in this study were primary human cells. The cells used in this study are all primary adult human cells from DIEP or lipectomy procedures. The methods for derivation of cultured human perivascular stem cells (PerSCs) in this study are described in the methods, and as indicated in text of previous publications (Crisan et al., 2008, Donnelly et al., 2023). Patient samples were taken as part of routine clinical care. CD34+ bone marrow cells were acquired from Caltag Medsystems and STEMCELL technologies or from the bone marrow aspirates of patients undergoing joint replacement surgery.

Authentication: Authentication of PerSCs is described in Crisan et al 2008 Cell Stem Cell. All PerSC lines were routinely tested for relevant markers by flow cytometry (CD146, CD34, CD45, CD31).

Mycoplasma contamination: PerSC and CD34+ cells used in this study have tested negative for mycoplasma contamination.

Commonly misidentified lines (See ICLAC register): No commonly misidentified lines were used in this study.

Animals and other research organisms

Policy information about studies involving animals; ARRIVE guidelines recommended for reporting animal research, and Sex and Gender in Research

Laboratory animals: NOD-RAG-gc–/– mice constitutively expressing human IL3, GMCSF and Steel factor (NRG-3GS82) were used as hosts (8 -10 wk old) for transplantation. Mice were housed at ambient temperature (19-22C) with relative humidity of 45-65%, and a 12h-12h light-dark cycle.

Wild animals: No wild animals were used in this study.

Reporting on sex: All mice used were male as sample size was small to reduce variation.

Field-collected samples: No field-collected samples were used for this study.

Ethics oversight	All mice were housed at the Beatson Research Unit (University of Glasgow, UK). Experimental protocols for working with animals were approved by the local AWERB committee and national Home Office (PD6C67A47).

Note that full information on the approval of the study protocol must also be provided in the manuscript.

Plants

Seed stocks	n/a
Novel plant genotypes	n/a
Authentication	n/a

Flow Cytometry

Plots

Confirm that:

☒ The axis labels state the marker and fluorochrome used (e.g. CD4-FITC).

☒ The axis scales are clearly visible. Include numbers along axes only for bottom left plot of group (a 'group' is an analysis of identical markers).

☒ All plots are contour plots with outliers or pseudocolor plots.

☒ A numerical value for number of cells or percentage (with statistics) is provided.

Methodology

Sample preparation	PerSCs were harvested from human adipose tissue via enzymatic digestion and FACS as described in methods. Human CD34+ cells were acquired from BM samples, STEMCELL or CalTag Medsystems, after culture in models samples were collected using collagenase as described in methods.
Instrument	BD FACSAria cell sorter (BD Biosciences), BD FACSCanto ll (BD Biosciences).
Software	Collection: FACSDiva v8.0 (BD Biosciences).<br>Analysis: FlowJo software v10.5.3 (FlowJo).
Cell population abundance	At least 5x10e3 events were collected for each condition.
Gating strategy	For PerSC phenotyping in Figure 3f a gate was drawn on FCS-A/SSC-A to select the cell population, from this MFI values were obtained for each marker compared to an unstained control. For Figure 7b Forward (FSC-A) versus side scatter area (SSC-A) plots were used to identify viable cells; FSC-A versus FSC width (FSC-W) plots were used to identify single cells. A gate was added on CD45+ve cells to exclude any PerSCs and 3 gates were used to identify CD34+ve/CD38-ve, CD34+ve/CD38+ve and CD34-ve/CD38+ve populations. The CD34+ve/CD34-ve population represents both LT- and ST-HSCs, whereas the CD34+ve/CD38+ve and CD34-ve/CD38+ve populations represent haematopoietic stem and progenitor cells and committed progenitors, respectively. For FACS sorting CD34+ cells to seed LTC-IC assay and in vivo assay, FSC-A versus SSC-A plot used to identify viable cells; gate added on CD45+ve cells to remove any PerSCs; Lin-ve gate used to remove any committed progenitors and final gate added on CD34+ve population.<br>Other detailed gating stratgies for an extended marker panel for ST-HSC vs LT-HSC is shown in supplementary figure 12. For in vivo analysis, the gating strategy is shwn in detail in supplementary figure 13b i & ii.

☒ Tick this box to confirm that a figure exemplifying the gating strategy is provided in the Supplementary Information.
